# Alterations in gut microbiome and metabolomics in chronic hepatitis B infection-associated liver disease and their impact on peripheral immune response

**DOI:** 10.1080/19490976.2022.2155018

**Published:** 2022-12-15

**Authors:** Yue Shen, Sheng-Di Wu, Yao Chen, Xin-Yue Li, Qin Zhu, Kiyoko Nakayama, Wan-Qin Zhang, Cheng-Zhao Weng, Jun Zhang, Hai-Kun Wang, Jian Wu, Wei Jiang

**Affiliations:** a Department of Gastroenterology & Hepatology, Zhongshan Hospital of Fudan University, Shanghai, China; b Shanghai Institute of Liver Diseases, Fudan University Shanghai Medical College, Shanghai, China; c Department of Gastroenterology& Hepatology, Zhongshan Hospital Xiamen Branch of Fudan University, Xiamen, China; d Department of Emergency Medicine, Zhongshan Hospital of Fudan University, Shanghai, China; e Department of Medical Microbiology & Parasitology, MOE/NHC/CAMS Key Laboratory of Medical Molecular Virology, School of Basic Medical Sciences, Fudan University Shanghai Medical College, Shanghai, China; f CAS Key Laboratory of Molecular Virology and Immunology, Institute Pasteur of Shanghai, Chinese Academy of Sciences, Shanghai, China

**Keywords:** Chronic hepatitis B virus infection-associated liver disease, cirrhosis, antiviral treatment, microbiome, metabolome, peripheral immunity

## Abstract

Gut dysbiosis has been reported in chronic hepatitis B (CHB) infection, however its role in CHB progression and antiviral treatment remains to be clarified. Herein, the present study aimed to characterize gut microbiota (GM) in patients with chronic hepatitis B virus infection-associated liver diseases (HBV-CLD) by combining microbiome with metabolome analyses and to evaluate their effects on peripheral immunity. Fecal samples from HBV-CLD patients (n = 64) and healthy controls (n = 17) were collected for 16s rRNA sequencing. Fecal metabolomics was measured with untargeted liquid chromatography-mass spectrometry in subgroups of 58 subjects. Lineage changes of peripheral blood mononuclear cells (PBMCs) were determined upon exposure to bacterial extracts (BE) from HBV-CLD patients. Integrated analyses of microbiome with metabolome revealed a remarkable shift of gut microbiota and metabolites in HBV-CLD patients, and disease progression and antiviral treatment were found to be two main contributing factors for the shift. Concordant decreases in *Turicibacter* with 4-hydroxyretinoic acid were detected to be inversely correlated with serum AST levels through host-microbiota-metabolite interaction analysis in cirrhotic patients. Moreover, depletion of *E.hallii group* with elevated choline was restored in patients with 5-year antiviral treatment. PBMC exposure to BE from non-cirrhotic patients enhanced expansion of T helper 17 cells; however, BE from cirrhotics attenuated T helper 1 cell count. CHB progression and antiviral treatment are two main factors contributing to the compositional shift in microbiome and metabolome of HBV-CLD patients. Peripheral immunity might be an intermediate link in gut microbe-host interplay underlying CHB pathogenesis.

## Legend for graphical abstract

Integrated analyses of microbiome with metabolomics revealed a remarkable shift of gut microbiota and metabolites in HBV-CLD patients. Disease progression and entecavir treatment were found to be two main contributing factors for the shift. Novel host-microbiota-metabolite interplay was investigated (red, positive correlation; blue, negative correlation). *Ex vivo* results showed that exposure of PBMCs to BE from non-cirrhotic patients promoted expansion of T helper 17 cells whilst BE from cirrhotic patients attenuated T helper 1 cell count, suggesting peripheral immunity may be one of mechanisms by which overall bacterial products exert profibrotic effects and have an impact on prognosis of HBV-CLD patients. Our research confers new insights into the role of gut dysbiosis and metabolomics in the pathogenesis of HBV-CLD, and underscores that disrupted peripheral immunity homeostasis during the microbe-host interplay may contribute to fibrosis progression in HBV-CLD. CHB, chronic hepatitis B (treatment-naive); Crrh, cirrhosis; ETV, entecavir; HBV-CLD, chronic hepatitis B virus infection-associated liver diseases; HCs, healthy controls; MCFAs, medium chain fatty acids; NC, non-cirrhosis; Th1, T helper 1; Th17, T helper 17.

**Abbreviations**: ALB, albumin; ALP, alkaline phosphatase; ANOISM, analysis of similarities; AST, aspartate aminotransferase; BE, bacterial extracts; BMI, body mass index; CC, compensated cirrhosis; CHB, chronic hepatitis B; DB, direct bilirubin; DC, decompensated cirrhosis; DCA, deoxycholic acid; ETV, entecavir; FDR, false discovery rate; GGT, γ-glutamyl transpeptidase; GM, gut microbiota; HBV, hepatitis B virus; HBV-CLD, chronic hepatitis B virus infection-associated liver diseases; HCs, healthy controls; HCC, hepatocellular carcinoma; LC-MS, liquid chromatography-mass spectrometry; LRE, liver-related events; LS, liver stiffness; ImP, imidazole propionate; IQR, interquartile range; MCFAs, medium chain fatty acids; OCT, organic cation transporter; OPLS-DA, orthogonal partial least square discriminant analysis; PBMCs, peripheral blood mononuclear cells; PERMANOVA, permutational multivariate analysis of variance; PLS-DA, partial least square discriminant analysis; PCA, principal component analysis; PcoA, principal coordinates analysis; PT, prolonged prothrombin time; SDs, standard deviations; TB, total bilirubin; Tregs, regulatory T cells; Th1, T helper 1; Th17, T helper 17.

## Graphical Abstract

**Figure:**
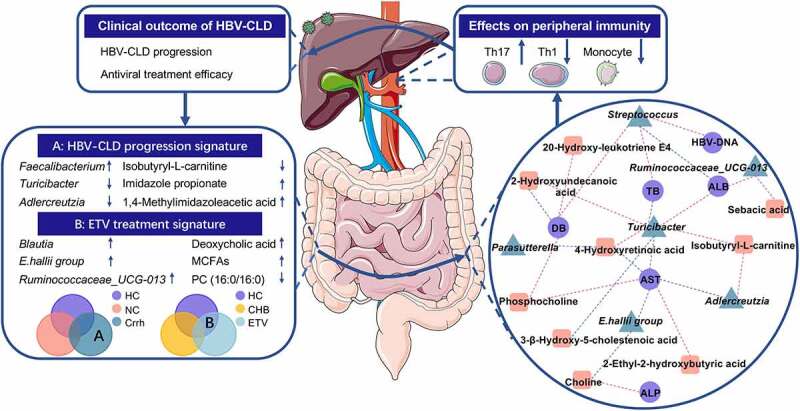

## Introduction

Globally, there are still more than 257 million hepatitis B virus (HBV)-positive subjects.^1^ More than 90 million of HBV infection exists with progression of 30 million infected individuals to chronic hepatitis B (CHB) in China although HBV vaccination resulted in a significant reduction in HBV infection in general public.^2^ The chronicity of HBV infection is a global health burden and the leading cause for liver-related events (LREs) including decompensation, hepatocellular carcinoma (HCC), liver transplantation, and liver-related mortality.^3^ Nucleoside/nucleotide analogs and pegylated interferon-α (PEG-IFN-α), the first‐line anti‐viral mediations, have slowed down CHB progression with reduced LREs.^4–6^ However, progression undergoes even if a viral load is undetectable (<50 IU/ml) in a small fraction of subjects.^7^ Therefore, pathophysiologic mechanisms of the CHB progression remain not fully understood.

The influence of gut microbiota (GM) and its derivatives on liver pathophysiology has drawn great attention.^8,9^ Based on the structural link to the intestine, the liver receives various gut-derived substances (bacterial products, environmental toxins, and food antigens) via the biliary tract, portal vein, and systemic circulation. Considering the uniquely intertwined connection between gut and liver, the liver plays a vital role in the balance of foreign substances and the systemic milieu.^10^ Indeed, significant changes in GM composition were determined in CHB patients characterized by a gain in bacteria including *Firmicutes, Streptococcus, Proteobacteria*, and *Prevotella*.^11–14^ HBV infection has been demonstrated to alter the composition of the GM in a mouse model by hydrodynamic injection to mimic either acute or chronic HBV infection.^15^ In addition, entecavir (ETV) treatment effectively corrected GM dysbiosis developed in persistent HBV-infected mice.^16^ Nonetheless, detailed genera profile alterations in connection with disease progression and prognosis under antiviral treatment have not been systematically established.

The profiling of complex microbial communities using 16s rRNA sequencing lacks quantitative functional annotation, which could be supplemented by microbe-derived metabolites. Fecal metabolomics appears to be a novel tool to explore the interplay between the host and GM since metabolomic data may explain impacts of GM on host metabolic and immunogenic alterations.^17,18^ Growing evidence revealed that gut microbe-derived metabolites, such as trimethylamine, bile acids, short-chain fatty acids, significantly affect the initiation and progression of liver diseases.^19–21^ Therefore, comprehensive integration of host-microbiota with metabolomic data may provide a clue to decipher the altered homeostasis of GM in CHB pathogenesis. In addition to intestinal function and host metabolism, gut microbe-derived metabolites are able to regulate host immune system.^22,23^ Peripheral immunity influences the course of chronicity, antiviral response as well as the prognosis of chronic hepatitis B virus infection-associated liver diseases (HBV-CLD).^1,24–28^ From clinical observations, it is learnt that persistent inflammatory response may upregulate immunosuppressive pathways and promote recruitment of immune regulatory cells to sustain chronic HBV infection in an immune-tolerant state.^1,24,25^ In this context, convincing evidence has suggested that balances of Th1/Th2 and Treg/Th17 cells orchestrate HBV clearance and liver inflammation.^26–28^ Thus, exploring the direct and indirect immunomodulating effects of GM on peripheral immunity creates a unique opportunity to develop both novel prognostic signature and therapeutic approaches for HBV-CLD.

Therefore, in the present study, GM dysbiosis in HBV-CLD patients was characterized by employing 16S rRNA gene amplicon sequencing and liquid chromatography-mass spectrometry (LC-MS). Microbiome and metabolomic profile in association with liver stiffness regression after 5-year ETV treatment has been established. Novel host-microbiota-metabolite interplay underlying CHB pathogenesis was investigated. Moreover, bacterial extracts (BE) from both HBV-CLD patients and healthy controls (HCs) were prepared for *ex vivo* exposure to confirm the effects of HBV-CLD-associated GM on the peripheral immune response. The findings confer new insights into the role of GM in the pathogenesis of HBV-CLD, and underscore that peripheral immunity may act as an intermediate link in the microbe-host interplay affecting CHB pathogenesis.

## Materials and methods

### Patients and study design

A total of 107 fecal samples were collected and 81 subjects were enrolled for final inclusion that was divided into two groups: HBV-CLD patients (n = 64) and HCs (n = 17). Patients with entecavir-based treatment for 104 weeks were recruited from two multicenter, randomized, and controlled trials registered at ClinicalTrials.gov (NCT01938781, NCT01938820). Treatment-naïve patients were recruited from the outpatients of Zhongshan Hospital of Fudan University from January 2015 to June 2016. HCs were recruited from volunteers for routine health examinations in Zhongshan Hospital. Key inclusion criteria were as follows: (1) HBsAg-positive status lasted for at least 6 months before screening; (2) Serum HBV DNA level was higher than 20,000 IU/mL for HBeAg-positive patients or 2000 IU/mL for HBeAg-negative patients. Diagnosis of compensated cirrhosis was made according to: (1) Metavir fibrosis grade reached F4 based on liver biopsy; (2) Esophageal varices, excluding non-cirrhotic portal hypertension, was identified; (3) When biopsy and endoscopy were unavailable, two of the following four criteria were met: (1) Cirrhotic liver morphology was indicated by imaging modalities (including ultrasonography, computed tomography or magnetic resonance imaging); (2) Blood platelet count was <100 × 10^9^/L with no other explanation; (3) Albumin was <35 g/L, or international normalized ratio was >1.3; (4) Liver stiffness parameter was >12.4 kPa when alanine aminotransferase (ALT) was <5 upper normal limit. Hepatic decompensation was defined by the presence of ascites, gastric or esophageal varices bleeding, hepatic encephalopathy or spontaneous bacterial peritonitis. Patients with: (1) abnormal blood glucose/lipid, abnormal urine and stool; (2) other viral hepatitis, autoimmune liver diseases or malignancy; (3) previous use of antibiotics, probiotics or immunosuppressive drugs within 8 weeks was excluded. Child-Pugh score was calculated from 5 parameters (total bilirubin (TB), serum albumin, prothrombin time (PT), ascites and hepatic encephalopathy) to indicate the severity of chronic liver disease. Written informed consent was obtained from all patients enrolled in the study. The study protocol (B2017-192 (2)) was in accordance the ethical guidelines of the 1975 Declaration of Helsinki, and was approved by the ethics committee of Zhongshan Hospital of Fudan University.

### DNA extraction and 16S rRNA gene amplicon sequencing and analysis

All stool samples were freshly collected and frozen at −80°C within 2 h after sampling. DNA was extracted using the E.Z.N.A. soil DNA kit (Omega Bio-tek, Norcross, GA, U.S.). The hypervariable region V3-V4 of the bacterial 16S rRNA gene was amplified and sequenced using MiSeq platform (Illumina, San Diego, California, USA). Raw reads were deposited into the NCBI sequence read archive database (Bio-Project ID: PRJNA771991). 16S rRNA sequencing data were processed using quantitative insights into microbial ecology (QIIME2 V.2020.2) software. After paired-end merging and error correction, DADA2 was further applied for quality filtration, and a total of 663147 and 2470462 sequences were obtained from HCs and HBV-CLD patients, respectively. After subsampling each sample to an equal sequencing depth (21107 reads per sample), qualified sequences were clustered into 706 de novo operational taxonomic units at the 97% similarity threshold level. A total of 230 genera of 11 phyla were subsequently identified by RDP Classifier against Silva database (V.13.8). Alpha diversity analysis was undertaken by Mothur. Principal co-ordinates analysis (PCoA) and partial least square discriminant analysis (PLS‐DA) were utilized to evaluate global microbiota composition (β-diversity) based on Bray-Curtis distances with statistical differences between groups calculated by analysis of similarities (ANOISM) and permutational multivariate analysis of variance (PERMANOVA).

### Fecal metabolome profiling and analysis

Fecal metabolome profiling of 39 samples was performed on a Thermo UHPLC system coupled with a Thermo Q Exactive Mass Spectrometer equipped with an electrospray interface. Fecal samples were accurately weighed following homogenization and ultrasonication. Quality control samples (n = 7) were pooled into vials in which aliquots of each sample (10 μL) were mixed together for later LC/MS analysis together with patient samples.

Metabolomic data were log10 transformed and analyzed by the progenesis QI software for peak detection, extraction, alignment, and integration (Majorbio Bio-Pharm Technology Co. Shanghai). In total, 28118 and 32587 peaks were detected in positive and negative modes. Metabolites were further annotated against public databases including Kyoto Encyclopedia of Genes and Genomes, the Human Metabolome Database, Lipid Maps (v2.3) and METLIN. Principal component analysis (PCA) was carried out for ruling out samples outside 95% confidence interval. Orthogonal partial least square discriminant analysis (OPLS-DA) was performed to visualize metabolic alterations between groups based on response permutation testing (n = 200). In general, metabolites with variable importance in the projection (VIP) >1, (2) fold change (FC) >1.2 or <0.83 and (3) *p_fdr_* <0.05 were considered differentially abundant between groups.

### Preparation of bacterial extract from fecal samples

BE was prepared as previously described.^29,30^ Fecal sample at 150 mg was fully suspended in 3 mL of PBS and subsequently filtrated through a 40-μm-cell strainer for three times. Isolated bacteria were washed twice with 1.5 mL of PBS, and then resuspended in PBS supplemented with protease inhibitor and phosphatase inhibitor. After heat-inactivation at 65°C for 1 hour and sonication for 10 min, protein concentration and LPS levels were measured separately with BCA protein assay and an endotoxin detection kit. All BE preparations were adjusted to a protein concentration of 5 mg/mL, as a concentration higher than this level led viability of peripheral blood mononuclear cells (PBMCs) to be reduced to <80% (**Fig. S5E**). A final BE concentration was subsequently normalized to LPS levels <1 ng/ml.

### Isolation and exposure of human PBMCs to bacterial extract

PBMCs were isolated from HCs as previously described and stored in fetal bovine serum containing 10% DMSO at −80°C.^31^ Cell counting and viability were examined with trypan-blue staining. After being washed twice with PBS, PBMCs were seeded in a density of 3*10E6 cells/mL in RPMI medium supplemented with 10% FBS, 1% penicillin/streptomycin and 1% glutamine.

An *ex vivo* model was applied to stimulate PBMCs with BE from HCs, untreated non-cirrhotic and cirrhotic CHB patients mimicking the pathological circumstance under which the intestinal permeability is increased and bacteria along with pathogen-associated molecular patterns translocated into the mesenteric portal blood flow. Subsets of T helper 1 (Th1), T helper 17 (Th17) and regulatory T (Treg) cells were induced for differentiation 72 hrs after BE exposure with corresponding antibodies and cytokines (Th1: anti-human CD3 (2 µg/mL), anti-human CD28 (2 µg/mL), recombinant human IL-2 (10 ng/mL), anti-human IL-4 (10 µg/mL) and recombinant human IL-12 (10 ng/mL); Th17: anti-human CD3 (2 µg/mL), anti-human CD28 (5 µg/mL), anti-human IFN-γ (5 µg/mL), anti-human IL-4 (5 µg/mL), recombinant human IL-6 (30 ng/mL), recombinant human TGF-β (3 ng/mL), recombinant human TNF-α (10 ng/mL) and recombinant human IL-1β (10 ng/mL); Treg: anti-human CD3 (2 µg/mL), anti-human CD28 (2 µg/mL), recombinant human IL-2 (5 ng/mL), TGF-β (3 ng/mL)). Afterward, fresh medium supplemented with cell stimulation cocktail (2 μL/ml, including PMA, ionomycin, brefeldin A and monensin) in the absence of BE was given for 4 h. Undifferentiated PBMCs were stimulated for 3 days in the presence of BE.

### Flow cytometry of human PBMCs

Cells were harvested after BE exposure for subsequent surface antigen staining after blocking the Fc receptor. Following surface antibodies were used: CD3-PE, CD3-FITC, CD4-Percp, CD8-PECy7, CD14-BV421, CD19-PECy7, CD25-APC, CD56-FITC, CD45RO-APC, HLA-DR-APC, and CCR7-BV421. Fixation/permeabilization solution kit was employed to stain intracellular cytokines. Following intracellular antibodies were used: IFN-γ-BV510, IL-17A-BV605, FoxP3-BV421, and IL-10-PECy7. Stained PMBCs were analyzed by flow cytometry in BD LSR Fortessa, and data were further analyzed via Flow Jo software V10 (Tree Star). Detailed gating strategies of target cell population were shown in **Fig. S4** and **Fig. S5**.

### Statistical analysis

All data analysis was performed by SPSS version 22.0 software (SPSS Inc., Chicago, IL, United States) and R (V.4.0.2). Continuous variables are presented as means ± standard deviations (SDs) or median with interquartile range (IQR), as appropriate. Statistical analysis of continuous variables assessing clinical characteristics with normal distribution was calculated using two-tailed independent *t* test or ANOVA test. Non-parametric Mann-Whitney U-test or Kruskal-Wallis test was applied for the comparison of data that did not fit a normal distribution. Categorical variables were compared by the Chi-square test. Partial spearman rank correlation test was used to analyze the correlation between genus and metabolites. Correlations of clinical characteristics with taxa/metabolites were assessed as well. A *p* value adjusted using Benjamini-Hochberg false discovery rate (FDR) <0.05 was regarded as statistically significant.

## Results

### Clinical characteristics of the enrolled subjects

**Figure 1:**
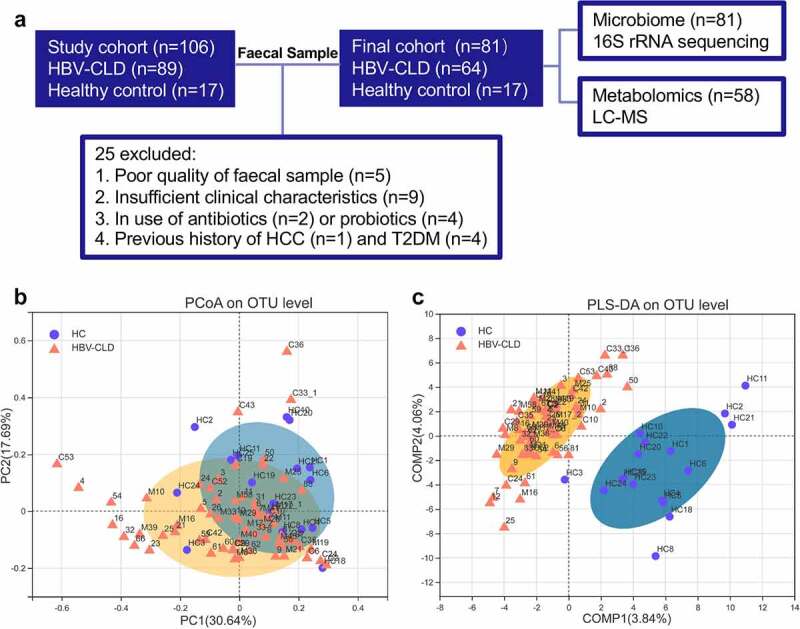
:Distinct fecal microbiota profiles of HBV-CLD patients and HCs. Flow chart of the study design (a); β diversity (based on Bray-Curtis distances) evaluated by PCoA analysis (b); PLS-DA analysis (c). HBV-CLD, chronic hepatitis B virus infection-associated liver diseases; HC, healthy control; HCC, hepatocellular carcinoma; LC-MS, liquid chromatography–mass spectrometry; T2DM, diabetes mellitus type 2.

16s rRNA gene amplicon sequencing was finally performed in a total of 81 fecal samples, and untargeted metabolomic profiling was undertaken in a subset of 39 samples (Figure 1a). Number of CHB patients diagnosed with and without cirrhosis was 41 and 23, respectively. Demographic characteristics of these cohorts are shown in **Table 1**. Body mass index (BMI) was balanced across all groups. Cirrhotic patients seemed to be older than non-cirrhotic subjects (median: 52 vs. 47) with no statistical significance. Cirrhotic patients had significantly higher levels of serum aspartate aminotransferase (AST), total bilirubin (TB), direct bilirubin (DB), prolonged prothrombin time (PT) and increased international normalized ratio with significantly decreased platelet, erythrocyte, leukocyte counts, reduced albumin (ALB), and cholinesterase levels than non-cirrhotic patients. Serum γ-glutamyl transpeptidase (GGT) and alkaline phosphatase (ALP) levels tended to be higher in cirrhotic patients than non-cirrhotic subjects although statistical analysis did not reach any significance.

### Microbial community diversity of HBV-CLD patients

Alpha diversity indexes indicating community richness, diversity, evenness and coverage were assessed via Sobs, ACE, Chao, Shannon, Simpson, Shannoneven, Simpsoneven, and Coverage indexes. No significant changes in α diversity was observed in the comparison between HBV-CLD patients and HCs. Both qualitative PCoA and PLS‐DA analyses were performed to evaluate β diversity. Overall GM community of HBV-CLD patients was different from that of HCs as indicated by PCoA analysis (*p* < .01, PERMANOVA test, Figure 1b) and PLS-DA analysis (Figure 1c). Detailed subgroup analyses of α diversity and β diversity are shown in **Supplementary file 1**.

ANOSIM analysis was further performed. Overall, differences in GM between the predefined groups were small but statistically significant with the highest differences observed between HCs and cirrhotic subjects (**Table 2**). Notably, in the comparison of HCs and non-cirrhotic patients, there was a marked difference in overall microbial community between HCs and treatment-naive patients instead of those with antiviral treatment (**Table 2**). In spite of receiving antiviral treatment or not, the difference in overall microbial community between HCs and the respective cirrhotic patients were of statistical significance (**Table 2**). In the comparison between cirrhotic and non-cirrhotic patients, significant difference was confirmed in those receiving antiviral treatment while not in treatment-naïve patients (**Table 2**). In summary, significant alterations of gut microbial homeostasis were confirmed during CHB progression. Antiviral therapy appeared not only to inhibit HBV replication but also partially corrected gut dysbiosis, and this phenomenon seemed to be more distinct in non-cirrhotic patients than cirrhotic patients.

### Overall microbiota distribution and abundance

**Figure 2:**
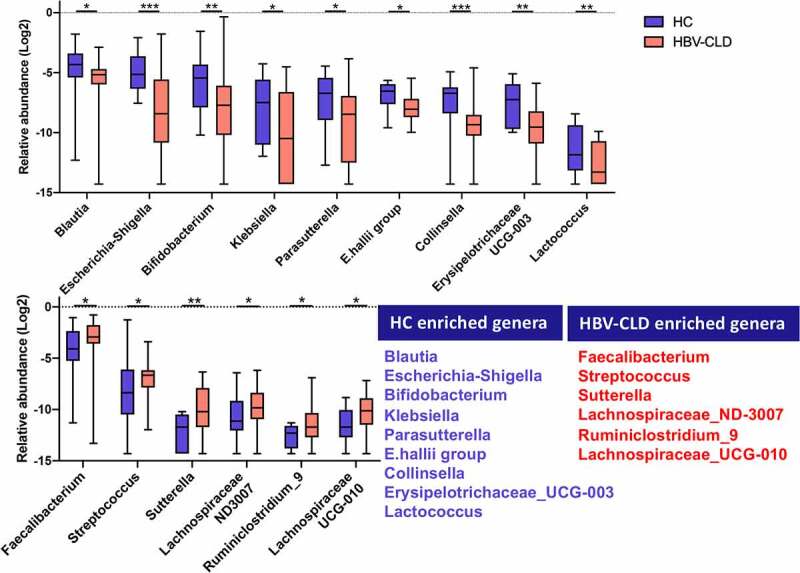
:Gut microbiota profile in HBV-CLD patients. The abundance of genera was log-transformed with 0 values assigned with 1e-05. Box plots indicate median (middle line), 25th, 75th percentile (box) and maximum and minimum values (whisker). *p_fdr_<0.05, **p_fdr_<0.01, ***p_fdr_<0.001, ****p_fdr_<0.0001. HBV-CLD, chronic hepatitis B virus infection-associated liver diseases; HC, healthy control.

**Figure 3:**
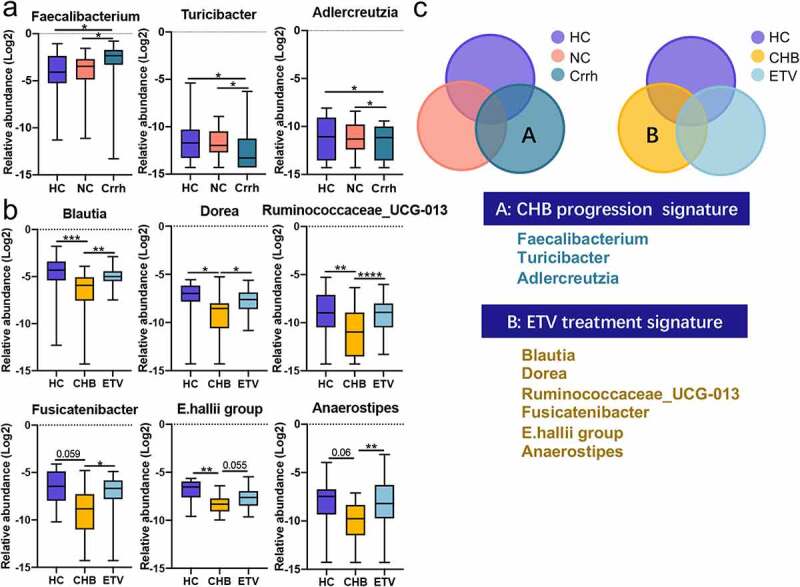
:Distinct gut microbiota profile regarding disease progression and antiviral treatment. Genera correlated with disease progression (a); antiviral treatment (b); Venn diagram outlined the genera associated with disease progression and antiviral treatment (c). The abundance of genera was log-transformed with 0 values assigned with 1e-05. Box plots indicate median (middle line), 25th, 75th percentile (box) and maximum and minimum values (whisker). *p_fdr_<0.05, **p_fdr_<0.01, ***p_fdr_<0.001, ****p_fdr_<0.0001. CHB, chronic hepatitis B (treatment naive); Crrh, cirrhosis; ETV, entecavir; HC, healthy control; NC, non-cirrhosis.

Relative abundance analysis was performed based on 11 phyla, 20 classes, 32 orders, 65 families and 230 genera detected in all 81 fecal samples. Distribution of predominant bacteria at different taxonomic levels is shown in **Supplementary file 1**. Relative genus abundance was compared between HCs and HBV-CLD patients (*p_fdr_*<0.05, Mann-Whitney U-test). Nine genera (*Blautia, Escherichia-Shigella, Bifidobacterium, Klebsiella, Parasutterella, E.hallii group, Collinsella, Erysipelotrichaceae_UCG-003, Lactococcus*) were enriched in HC subjects; whereas six genera (*Fecalibacterium, Streptococcus, Sutterella, Lachnospiraceae_ND-3007, Ruminiclostridium 9, Lachnospiraceae_UCG-010*) were markedly increased in HBV-CLD patients (Figure 2). Genus that was found to correlate with disease progression as well as antiviral treatment according to subgroup analyses was present in **Table. S1& Table. S2** (*p_fdr_* <0.05, Kruskal-Wallis test). Twenty-one genera reached statistical significance in the subgroup analysis based on the presence of cirrhosis (**Table. S1**). The relative abundance of *Fecalibacterium* was similar between HCs and non-cirrhotic patients; whereas it was increased in cirrhotic patients, indicating that its abundance was positively correlated with disease progression of CHB. In contrast, the abundance of *Turicibacter* and *Adlercreutzia* exhibited a negative correlation with disease progression (Figure 3a). In addition, the abundance of *Bifidobacterium, Escherichia-Shigella*, and *Sutterella* in HBV-CLD patients regardless of the stage of fibrosis was shown to be at levels similar to HCs (Figure 3b). In addition, thirty-seven genera reaching suggestive statistical differences in subgroup analysis based on antiviral treatment were identified (**Table. S2**). Of note, the relative abundance of *Blautia, Dorea*, and *Ruminococcaceae_UCG-013* remained similar between HCs and CHB patients receiving antiviral treatment; whereas it was significantly different from those without treatment, indicating that the antiviral treatment may correct the gut dysbiosis (Figure 3c). The abundance of *Fusicatenibacter, E.hallii group* and *Anaerostipes* displayed the same signature while with the adjusted *p* value to be 0.059, 0.055 and 0.060 in the comparison between HCs and non-cirrhotic patients (Figure 3c). However, the abundance of *Escherichia-Shigella, Collinsella*, and *Solobacterium* in HBV-CLD patients regardless of antiviral treatment was similar to that in HCs (Figure 3d). In summary, genera *Fecalibacterium, Turicibacter*, and *Adlercreutzia* were correlated with CHB progression, and changes in genera *Blautia, Dorea* and *Ruminococcaceae_UCG-013* were partially restored by ETV treatment (Figure 3e).

### Fecal metabolomic profile in HBV-CLD patients

#### Overall alteration of fecal metabolites in HBV-CLD patients

Untargeted metabolomics on thirty-nine fecal samples (HCs, n = 11, HBV-CLD, n = 28) was further performed considering the interplay between GM and host metabolism. OPLS-DA analysis indicated that metabolic composition of HBV-CLD patients was completely separated from that of HCs (E^−^R^2^Y (cum) = 0.994, Q^2^ (cum) = 0.708; E^+^ R^2^Y (cum) = 0.993, Q^2^ (cum) = 0.725, **Fig. S1A**). 2940 annotated metabolites were identified and 206 metabolites were statistically changed in HBV-CLD patients when compared to HCs (*p_fdr_*<0.05, **Table. S3**). These metabolites are involved in tryptophan metabolisms, primary bile acid biosynthesis, phenylalanine, tyrosine, and tryptophan biosynthesis, one carbon pool by folate, histidine metabolism, glycerophospholipid metabolism, cholinergic synapse, choline metabolism in cancer, central carbon metabolism in cancer, bile secretion (**Fig. S1D**).

**Figure 4:**
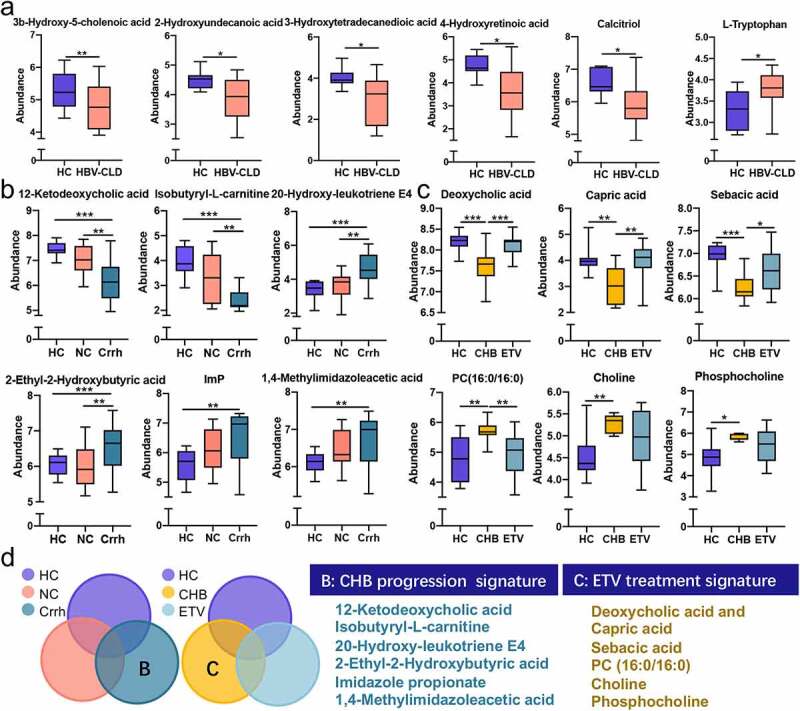
:Distinct fecal metabolome signature of HBV-CLD patients and HCs. Representative metabolites that were significantly changed in HBV-CLD (a); correlated with disease progression (b); antiviral treatment (c); Venn diagram outlined the metabolites associated with disease progression and antiviral treatment (d). The abundance of metabolites was log-transformed with 0 values assigned with 1e-05. Box plots indicate median (middle line), 25th, 75th percentile (box) and maximum and minimum values (whisker). *p_fdr_<0.05, **p_fdr_<0.01, ***p_fdr_<0.001, ****p_fdr_<0.0001. CHB, chronic hepatitis B (treatment naive); Crrh, cirrhosis; ETV, entecavir; HBV-CLD, hepatitis B virus related chronic liver diseases; HC, healthy control; NC, non-cirrhosis.

Representatively differential metabolites concerning bile acids, fatty acids, vitamins, and amino acids between HCs and HBV-CLD patients are present (Figure 4a). 3-β-Hydroxy-5-cholestenoic acid was dramatically decreased in HBV-CLD patients, and its plasma level was able to predict α diversity.^32^ 2-Hydroxyundecanoic acid, belonging to a group of medium chain fatty acids (MCFAs) that exert multiple effects including anti-fungal and anti-inflammatory, was decreased in HBV-CLD patients.^33,34^ Furthermore, reduction of long-chain fatty acids, such as 3-hydroxytetradecanedioic acid, which was down-regulated in the necrosis and apoptosis of hepatic L02 cells induced by Pekinenal, was also observed in HBV-CLD patients.^35^ Levels of calcitriol and 4-hydroxyretinoic acid were significantly lower in HBV-CLD patients than HCs. Vitamins A and D are able to shape GM community through regulation of intestinal epithelium and mucosal immune system.^36^ Their relevant metabolites are involved in the pathogenesis of HBV-CLD, thus indicating vitamins are likely to participate in molecular communications between microbiota and host.^37–39^ In contrast, L-tryptophan was significantly up-regulated.

#### Subgroup analysis of fecal metabolite shift in HBV-CLD patients

In consistent with contributing factors in gut microbial shift, OPLS-DA analyses based on disease severity **(Fig. S1B)** and antiviral treatment **(Fig. S1C)** revealed a complete separation of metabolites. Metabolites which were most distinct in cirrhotic patients compared to HCs and non-cirrhotic patients were fully explored (Figure 4b). 12-Ketodeoxycholic acid is considered as a major transient product in the 7α-dehydroxylation of cholic acid by 7-dehydroxylating bacteria, and it was significantly downregulated in cirrhotic patients^40.^ A decrease of isobutyryl-L-carnitine was most predominant in cirrhotic patients. In contrast, higher levels of 20-hydroxy-leukotriene E4 and 2-ethyl-2-hydroxybutyric acid were found in cirrhotic patients than non-cirrhotic patients and HCs. Meanwhile, metabolites involving histidine metabolism including imidazole propionate (ImP) 1,4-methylimidazoleacetic acid were elevated in cirrhotic patients compared to HCs. Microbe-derived ImP may contribute to elevated inflammatory tone in the mucosal lining and was associated with gastrointestinal inflammation.^41^ Recently, it has been shown to be involved in pathogenesis of type 2 diabetes while its role in liver diseases remains to be further clarified.^42,43^

Regarding subgroup analysis based on antiviral treatment, more attention was paid to metabolites in which difference was most pronounced in treatment-naïve patients compared to HCs and patients receiving antiviral treatment (Figure 4c). Deoxycholic acid (DCA) and MCFAs (capric acid, sebacic acid) were much lower in treatment-naïve patients than HCs and patients receiving ETV. In contrast, the level of fecal phosphatidylcholine PC (16:0/16:0) displayed an opposite trend. Increased level of PC (16:0/16:0) was reported in neoplastic tissues of HCC patients.^44^ In addition, choline and phosphocholine were more pronounced in untreated patients than HCs and ETV-treated patients as well.

Taken together, histidine-relevant metabolites, such as ImP and 1,4-methylimidazoleacetic acid, were enriched in cirrhotic patients, signifying its potential role in CHB progression. Although not prominent, the attenuation of choline metabolism in patients with ETV treatment compared to treatment-naïve individuals is noteworthy (Figure 4d).

### Taxonomic and functional composition of the gut microbial community and metabolites was correlated with hepatic dysfunction

### Correlations between the disease-linked microbiota and metabolites

**Figure 5:**
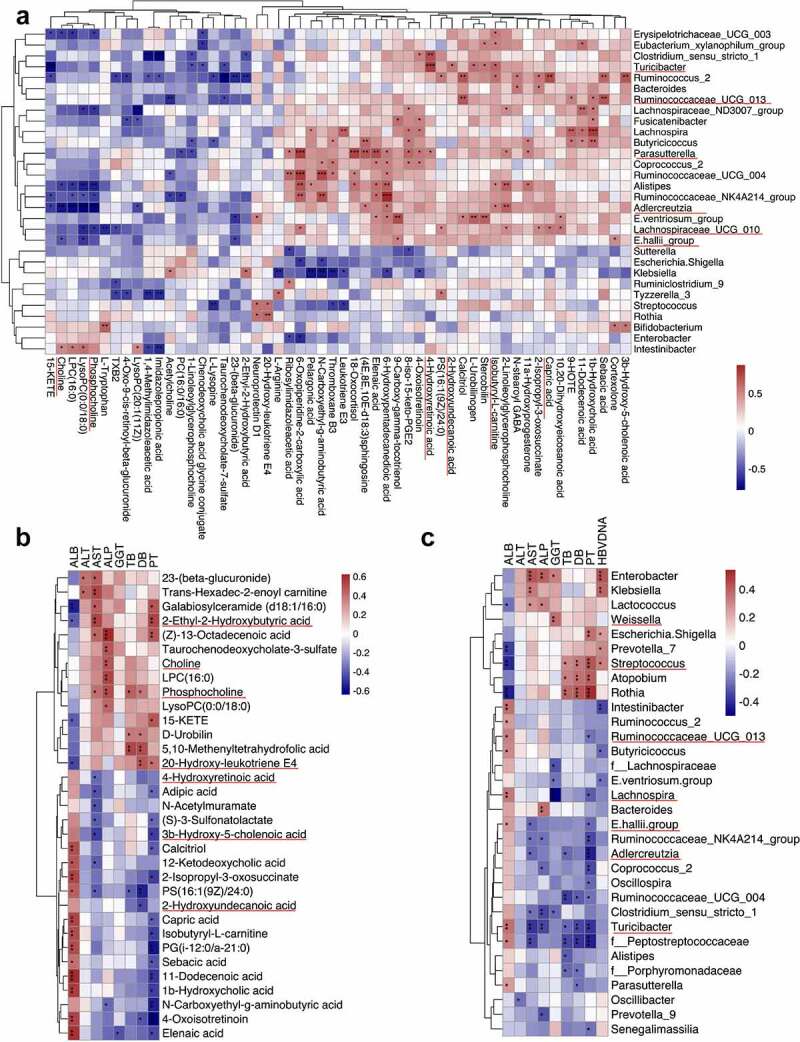
:The host-microbiota-metabolite interplay in HBV-CLD patients. The heatmap depicts correlation (partial spearman analysis) of disease-related taxa and metabolites (a); disease-related metabolites and clinical indexes (b); disease-related metabolites and clinical indexes (c). *p_fdr_<0.05, **p_fdr_<0.01, ***p_fdr_<0.001. ALB, albumin; ALT, alanine amino transferase; AST, aspartate aminotransferase; GGT, γ-glutamyl transpeptidase; TB, total bilirubin; DB, direct bilirubin; PT, prolonged prothrombin time.

A positive correlation was established between microbial genus and metabolites enriched in controls, as well as negative correlation between control-enriched genus and disease-enriched metabolites (Figure 5a). *Parasutterella*, the genus decreased in HBV-CLD patients, was positively correlated with 4-hydroxyretinoic acid (r = 0.543, *p* = .030). Reduced abundance of *Turicibacter* especially in cirrhotic patients was positively correlated with control-enriched metabolites, such as 2-Hydroxyundecanoic acid (r = 0.571, *p* = .021) and 4-hydroxyretinoic acid (r = 0.795, *p* = .000). Notably, *Turicibacter* (r = 0.621, *p* = .010) and *Adlercreutzia* (r = 0.536, *p* = .032) were positively correlated with isobutyryl-L-carnitine. A decreased level of sebacic acid was accompanied by reduced *Ruminococcaceae_UCG-013* (r = 0.703, *p* = .002) in treatment-naïve patients. An increased level of choline in treatment-naïve patients was negatively correlated with control-enriched genera *E.hallii group* (r = −0.508, *p* = .045).

### Correlations of microbial taxa and metabolites with clinical presentations

Partial spearman correlation tests were utilized to determine whether the alterations in fecal microbial and metabolic composition were correlated with biochemical parameters. Abundant metabolites were differentially fallen into two main clusters (Figure 5b). Decreased levels of 3-β-hydroxy-5-cholestenoic acid (r = −0.471, *p* = .013) and 4-hydroxyretinoic acid (r = −0.384, *p* = .048) were linked to increased AST. Moreover, 2-ethyl-2-hydroxybutyric acid (r = 0.524, *p* = .005) and phosphocholine (r = 0.418, *p* = .030) exhibited positive correlations with AST. In addition, DB level was positively linked to phosphocholine (r = 0.395; *p* = .041) and 20-hydroxy-leukotriene E4 (r = 0.495, *p* = .009); whereas negatively correlated with 2-Hydroxyundecanoic acid (r = −0.446, *p* = .020). A high level of choline especially in treatment-naïve patients was accompanied with increased ALP (r = 0.492, *p* = .009).

More importantly, novel accordance between taxa and host phenotypes was explored (Figure 5c). Increased AST was linked to low levels of *Turicibacter* (r = −0.386, *p* = .002), *Adlercreutzia* (r = −0.282, *p* = .029), and *E.hallii group* (r = −0.278, *p* = .032). Overabundant *Streptococcus* was positively correlated with serum TB (r = 0.301, *p* = .020), DB (r = 0.341, *p* = .008) and HBV-DNA copies (r = 0.327, *p* = .011); whereas negatively correlated with ALB (r = −0.403, *p* = .001). On the contrary, *Turicibacter* exhibited opposite correlations with TB (r = −0.286, *p* = .027), DB (r = −0.350, *p* = .006).) and ALB (r = 0.363, *p* = .004). Interestingly, *Ruminococcaceae_UCG-013* (r = 0.321, *p* = .012), which was depleted in treatment-naive patients while restored after ETV treatment, was positively correlated with ALB.

In summary, HC-enriched *Turicibacter* and 4-hydroxyretinoic acid concordantly presented an inverse correlation with serum AST. Key negative correlation was established between *E.hallii group* and choline, suggesting that novel host-microbiota-metabolite interplay may affect fibrosis progression and effects of antiviral treatment.

### Representative microbiota and metabolites reflected liver stiffness regression

The LS of patients receiving antiviral treatment was followed up for five years. LS value was defined as regression when it was decreased by more than 30% compared to the baseline. Twenty-five of 37 patients receiving ETV treatment achieved LS regression. Dynamic LS change in both regression and non-regression cohorts was shown in **Fig. S2A**. Four candidate genera including *E.hallii group, Blautia, R.torques group*, and *Coprococcus 3* in microbiota were found to be associated with LS regression (*p* < .05 while *p_fdr_*>0.05) **(Fig. S2B)**. Moreover, 143 metabolites exhibited remarkable difference between these two groups. Cholic acid was decreased; whilst DCA was increased in non-regression patients. Both L-glutamate and stearidonic acid were significantly increased in non-regression group **(Fig. S2C)**. Taken together, representative genera (*E.hallii group, Blautia*), and metabolites (cholic acid, DCA) were associated with LS regression, which highlights that specific gut dysbiosis may serve as potential prognosis markers in CHB patients with long-term antiviral treatment.

### Ex vivo *stimulation of patient-derived bacterial extracts on PBMCs*

**Figure 6:**
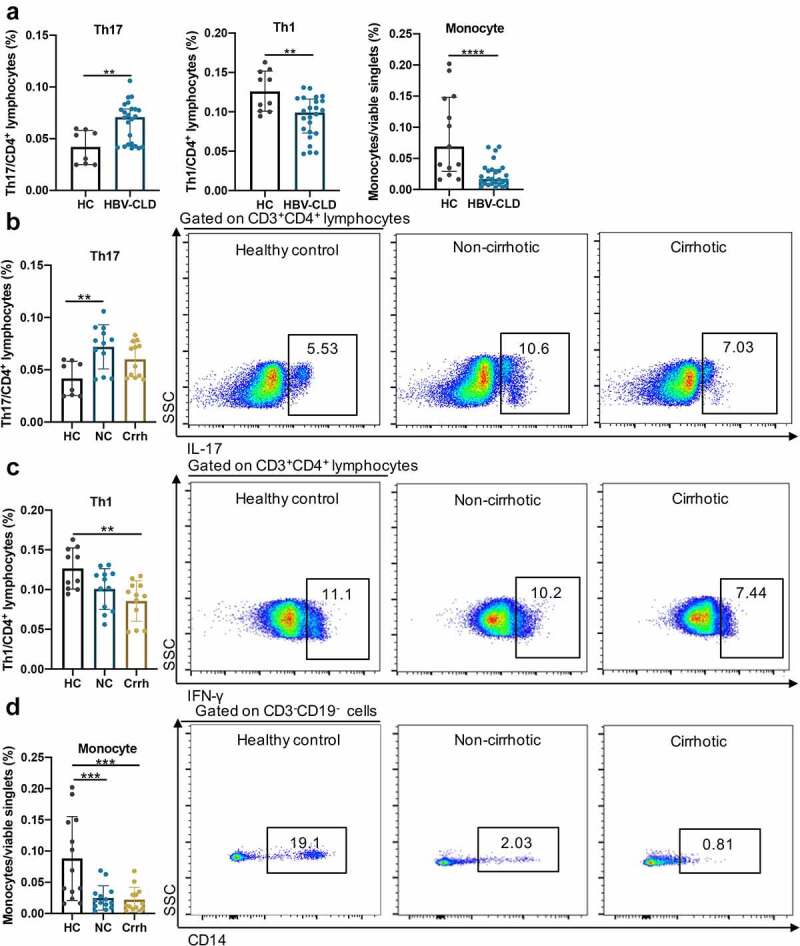
:Exposure of PBMCs to patient-derived BEs resulted in altered T cell subtype counts. Th17 (CD3^+^CD4^+^IL-17A^+^), Th1 (CD3^+^CD4^+^IFN-γ^+^), monocytes (CD3^−^CD19^−^CD14^+^) in response to BE exposure from HCs and HBV-CLD patients (a). Th17 (b), Th1 (c) and monocytes (d) in response to BE exposure from HCs, non-cirrhotic (NC) and cirrhotic (Crrh) patients. Data are shown as % of viable CD3^+^CD4^+^ lymphocytes for Th17 and Th1 cells, and % of viable singlets for monocytes. Box plots indicate individual values for each sample and median with interquartile range within groups. *p < .05, **p < .01, ***p < .001, ****p < .0001. BE, bacterial extracts; Crrh, cirrhosis; HBV-CLD, hepatitis B virus related chronic liver diseases; HC, healthy control; NC, non-cirrhosis; Th1, T helper 1; Th17, T helper 17.

In addition to the correlation analysis, direct effects of bacterial extracts (BE) from treatment naïve HBV-CLD patients on peripheral immunity remain unexplored, but fundamentally important. Therefore, PBMCs from HCs were exposed to BE from HBV-CLD cirrhosis (n = 24), non-cirrhosis (n = 12), and HC (n = 12) subjects. No significant difference was observed in total CD4^+^CD8^−^ cell (CD3^+^CD4^+^CD8^−^) counts after the treatment. Surprisingly, BE from treatment naïve HBV-CLD patients attenuated the expansion of T helper 1 cells (Th1 (CD3^+^CD4^+^IFN-γ^+^) p = .002), and this effect was remarkably predominant in patients with liver cirrhosis compared to HCs (Th1 p = .002, Figure 6a **and b**). In contrast, BE from HBV-CLD patients (p = .004), especially those without liver cirrhosis (p = .003), significantly provoked the expansion of T helper 17 cells (Th17 (CD3^+^CD4^+^IL-17A^+^)) compared to HCs (Figure 6a **and c**). The change of T regular cell count (Tregs (CD3^+^CD4^+^CD25^+^Foxp3^+^) p = .830) did not reach statistical significance (**Fig. S3A**) under BE exposure. Furthermore, there was a descending tendency in total CD8^+^ T cell count (CD3^+^CD4^−^CD8^+^) and a growing tendency in cytotoxic CD8^+^ T cells (CD3^+^CD4^−^CD8^+^CD45RO^−^CCR7^−^) counts after BE exposure from HBV-CLD patients (p > .05, **Fig. S3B&C**). In addition, antigen-presenting cell populations including monocytes (CD3^−^CD14^+^), myeloid dendritic cells (CD3^−^CD19^−^CD56^+^HLADR^+^) and B cells (CD3^−^CD19^+^), were also measured under e*x vivo* exposure to BE. Regardless of liver cirrhosis, BE from patients significantly attenuated expansion of monocytes compared to BE from HCs (p < .0001, (Figure 6a-d)). Further classifying monocytes based on relative expression levels of CD14 and CD16 surface proteins, our results indicated that pro-inflammatory intermediate (CD14^++^CD16^+^) monocytes were decreased by BE stimulation from patients compared to BE form HCs (Figure 2b). Accordingly, the ratio of classical monocytes (CD14^+^CD16^−^) increased after BE exposure from HBV-CLD patients in contrast to BE form HCs (data not shown). However, there was no change in B cells and dendritic cells under BE exposure. NK cell population (CD3^−^CD56^+^) was not changed strikingly however tended to decrease (**Fig. S3D**). Ratios of each cell population and statistical comparisons were present in **Table. S4**. In summary, BE from non-cirrhotic patients significantly enhanced the expansion of T helper 17 cells; however, BE from cirrhotic patients repressed T helper 1 cells, indicating that signaling from GM in addition to HBV-specific antigen may shape the peripheral immunity by altering T cell subpopulations.

## Discussion

In the present study, data generating from microbiome and metabolomic analysis have comprehensively demonstrated compositional changes of GM community in HBV-CLD patients, and identified distinct microbiota profiles and metabolomic signatures that were correlated to CHB progression and antiviral treatment. Furthermore, correlations between microbial and metabolomic profiles as well as between microbiota/metabolites and clinical presentations were extensively investigated. In addition, PBMCs were exposed to bacterial extracts from HCs and treatment-naïve CHB patients to examine the effects of HBV-CLD-associated microbiota and metabolites on the peripheral immunity, thus providing a new approach to investigate the role of gut microbiota in the pathogenesis of HBV-CLD.

In accordance with previous studies, the abundance of *Bifidobacterium* and *Escherichia-Shigella* was downregulated while overabundant *Streptococcus* was confirmed in HBV-CLD patients.^11,14,45,46^ Regarding host-microbe-metabolite interplay involved in disease progression, both *Turicibacter* and *Adlercreutzia* were found to positively correlate with isobutyryl-L-carnitine, which has been identified to be a biomarker of hepatic organic cation transporter-1.^47^ The three taxa and metabolites were decreased dramatically in cirrhotic patients compared to HCs. HC-enriched *Turicibacter* and *Adlercreutzia* have been proposed to be potentially beneficial genera with anti-inflammatory property.^48,49^ Indeed, both microbial species presented inverse correlation with AST, and *Turicibacter* exhibited a negative correlation with TB, DB, suggesting their beneficial roles in HBV-CLD.

Regarding the effects of antiviral treatment on GM, the depletion of *E.hallii group* and *Blautia* was confirmed in treatment-naïve patients; whilst it was restored in patients with ETV treatment compared to HCs. Both microbial genera tended to be decreased in a non-regression cohort. Butyrate-producing *E.hallii group*, which was inversely associated with AST and disease-enriched choline in this our study, has been proven to be beneficial in generating secondary bile acids.^50,51^ Considering the restored DCA level in patients with antiviral treatment compared to those without, a direct effect of antiviral treatment on the modulation of secondary bile acids through *E.hallii group* in the pathogenesis of HBV-CLD is worthy of further study. In addition, *Blautia*, a genus of anaerobic bacteria with probiotic characteristics, has drawn attention for its ability in the alleviation of metabolic syndrome as well as biological transformation.^52^ Interestingly, its decrease was confirmed both in patients with IgG4-related sclerosing cholangitis as well as in a mouse model of recombinant AAV-induced persistent HBV infection, suggesting its protective role in chronic liver disease.^16,53^ A prospective longitudinal cohort study indicated that the achievement of sustained virological response (SVR) restored the level of *Collinsella*, only in non-cirrhotic patients with chronic hepatitis .^54^ However, a lower level of the abundance of *Collinsella* was observed in HBV-CLD patients regardless of treatment compared to HCs. A longitudinal follow-up cohort study regarding the long-term effects of antiviral treatment on the GM community should be carried out in HBV-CLD patients.

In order to explore the mechanism by which the compositional and functional shift of GM contributed to the pathogenesis of HBV-CLD, further efforts were made to evaluate direct effect of GM from untreated HBV-CLD patients on peripheral immunity by exposure of PBMCs to BE treatment. BE from non-cirrhotic patients had the most capacity to elicit expansion of Th17 cells compared to HCs in consistent with increased frequency of circulating Th17 cells in CHB patients.^28^ Redundant Th17 immunity has been recognized as key immunopathological and prognostic elements responsible for fibrogenesis and progression to cirrhosis in CHB patients including hepatic stellate cell activation,^55,56^ increased expression of TGF-β,^57^ MMPs,^55,57,58^ collagen synthesis,^55,57^ and enhanced recruitment of inflammatory cells.^58,59^ Treg cells characterized by strong immunosuppressive activity play a key role in alleviating liver injury as well as sustaining persistent HBV infection via down-regulating inflammation depending on its presenting time. The ratio of Treg/Th17 has been suggested as an inflammation indicator of peripheral immunity in HBV-CLD patients.^27^ However, a slight increase in CD3^+^CD4^+^CD25^+^Foxp3^+^ Tregs in HBV-CLD patients did not reach statistical significance. These results suggested that in addition to HBV-related antigens, components in BE derived from non-cirrhotic HBV-CLD patients promoted a proinflammatory and profibrotic environment by inducing Th17 cells. In addition, BE from cirrhotic patients compared to HCs attenuated expansion of Th1 cells. Activation of Th1 immunity accompanied by the facilitation of HBV-specific cytotoxic T lymphocytes has been confirmed in CHB patients with successful elimination of HBV.^60,61^ Moreover, circulating Th1 in non-response CHB patients increased in a much lower degree than complete-response and part-response CHB patients during 52-week telbivudine monotherapy.^62^ Though with potent antiviral therapy, progression of liver fibrosis still occurs in 10%–20% of CHB patients with ambiguous risk factors.^7^ According to a previous report, GM plays a critical role in age-related immune clearance of HBV, and fecal microbiota transplantation may induce HBeAg clearance in a significant proportion of the patients with persistent positive HBeAg even after long-term antiviral treatment.^63,64^ The attenuation of Th1 cells induced by BE from cirrhotic patients provides a new hint regarding whether the effects of GM-derived signaling on peripheral immunity could be a novel factor contributing to the heterogeneity of long-term outcomes or treatment responses of antiviral therapy in CHB patients. Taken together, the major *ex vivo* findings of T cell subpopulation analysis demonstrated that patient-derived BE may shape peripheral immunity that contributes to fibrosis progression and has an impact on prognosis of HBV-CLD patients.

Several limitations should be acknowledged in our study. Firstly, small sample size of microbiome and metabolomics may restrict significance and stability of the results. A long-term follow-up of prospective cohorts is needed to further validate candidate taxa/metabolites. Secondly, 16s rRNA sequencing is not able to provide GM annotations accurate to “species” level. Vague annotations may lead to controversial results, for instance, the change in the abundance of Escherichia-Shigella varied from study to study.^11,46,65^ Thus, shotgun metagenomic sequencing should be performed for further microbiome analysis which is able to provide more accurate GM annotations and to give direct indications of GM function for further mechanistic investigations. Thirdly, absolute quantitation of candidate GM-derived metabolites should be obtained through targeted LCMS. The causal connection between microbiota and metabolite needs to be further verified through humanized animal models colonized by both candidate genera/metabolites and GM community isolated from HBV-CLD patients.^66^ All these limitations warrant further investigations with more adavnced technologies in expanded cohort size and extended follow-up duration.

In conclusion, disease progression (*Fecalibacterium, Turicibacter, Adlercreutzia*; isobutyryl-L-carnitine, ImP, 1,4-methylimidazoleacetic acid) and antiviral treatment (*Blautia, Ruminococcaceae_UCG-013, E.hallii group*; DCA, choline, phosphocholine) are major contributors to the compositional shift of gut microbiota and metabolites in HBV-CLD patients. The complicated interplay between altered genera and metabolites were fully identified through subsequent network of correlation analysis, which revealed a positive link between *Turicibacter* and 4-hydroxyretinoic and inverse correlation between *E.hallii group* and choline. Moreover, peripheral immunity may be one mechanism by which overall bacterial products exert profibrotic effects and have an impact on prognosis of HBV-CLD patients as non-cirrhotic patient-derived BE significantly provoked the expansion of T helper 17 cells; however, BE from cirrhotic patients repressed T helper 1 cells from PBMCs isolated from HCs. The findings confer new insights into the role of gut dysbiosis and metabolomics in the pathogenesis of HBV-CLD, and underscore that disrupted peripheral immunity homeostasis during the microbe-host interplay may contribute to fibrosis progression in HBV-CLD.

## Supplementary Material

Supplemental Material
