# Supplementary material for: Alterations in gut microbiome and metabolomics in chronic hepatitis B infection-associated liver disease and their impact on peripheral immune response
Source: Gut Microbes. 2022 Dec 15;15(1):2155018. doi: 10.1080/19490976.2022.2155018 (PMC9757487; doi:10.1080/19490976.2022.2155018)
Supplement: Supplemental Material [file KGMI_A_2155018_SM3018.zip › 10 supplementary file.docx]

## Supplementary Results

## Subgroup analysis of α diversity

Shannon index was found to be significantly lower and Simpson index was significantly higher in treatment-naive patients than those in HCs or patients receiving antiviral treatment **(Fig. S6A, Table. S5**). Moreover, Shannoneven and Simpsoneven indexes were significantly decreased in treatment-naive patients; whereas it was restored after antiviral treatment (**Fig. S6A, Table. S5**).

When cirrhotic patients were further divided into compensated cirrhosis (CC) and decompensated cirrhosis (DC) subgroups, Shannon and Shannoneven indexes in CC patients were statistically higher while Simpson index was lower than those with DC **(Fig. S6B)**. Child-Pugh score was negatively correlated with Shannon index; whilst it was positively correlated with Simpson index (**Fig. S6C**). In addition, there was a negative correlation between liver stiffness (LS) and indexes including Shannon, Shannoneven and Simpsoneven indexes; whereas the positive correlation was established between LS and Simpson index (**Fig. S6D**). In summary, these results suggested that the richness, diversity and evenness of gut microbial community were decreased during the progression of CHB. However, there was no significant difference in richness and coverage of microbiota community indicated by Sobs, ACE, Chao and coverage indexes regardless of disease progression and antiviral treatment (**Table S5**).

## Subgroup analysis of β diversity

Consistent with previous research, overall gut microbial community of CHB patients with or without cirrhosis was different from that of HCs as indicated by PCoA analysis (*p*<0.01, PERMANOVA test, **Fig. S7A**) and PLS‐DA analysis (**Fig. S7B**). Surprisingly, antiviral treatment was found to be another key driver of gut dysbiosis by either PCoA analysis (*p*<0.01, PERMANOVA test, **Fig. S7C**) or PLS-DA analysis (**Fig. S7D**). As shown in **Fig. S7C**, the structure of gut microbial community was altered substantially in treatment-naïve CHB patients when compared to HCs, and was corrected by antiviral treatment.

Furthermore, subsequent subgroup analysis within the cirrhotic group indicated that the gut microbial profile was not significantly different between those with CC and DC (*p*=0.138, PERMANOVA test, **Fig. S8A**) while it was significantly shifted by severity of liver disease as indicated by Child-Pugh Score (*p*=0.043, PERMANOVA test, **Fig. S8B**). Within the non-cirrhotic group, LS was likely to be correlated with the gut dysbiosis (*p*=0.059, PERMANOVA test, **Fig. S8C**). The overall gut microbial composition was not significantly shifted by BMI (*p*=0.084, **Fig. S8D**), age (*p*=0.120, **Fig. S8E**) and gender (*p*=0.616, **Fig. S8F**) (PERMANOVA test) of subjects enrolled in this study.

**The overall abundance and distribution of the predominant bacteria at different taxonomic levels**

The taxonomic distributions of the predominant microbiota (relative abundance >1% of the total sequences) among HCs, CHB patients with and without cirrhosis at phylum and genus levels are shown in **Fig. S9**. The 4 most predominant phyla were Firmicutes (58.69%), Bacteroidetes (31.70%), Proteobacteria (5.13%), Actinobacteria (3.62%), all together comprising 99.1% of the total sequences (**Fig. S9A**). The most prevalent genera were Bacteroides (19.74%), Faecalibacterium (16.04%), Prevotella_9 (8.87%), E.reclate (4.48%), Blautia (4.04%), Bifidobacterium (3.05%), Subdoligranulum (2.87%), Megamonas (2.77%), Escherichia-Shigella (2.70%), Lachnoclostridium (2.26%), Streptococcus (1.97%), Roseburia (1.81%), Phascolarctobacterium (1.56%), R.torques group (1.52%), Veillonella (1.26%), R.gnavus (1.02%), Fusicatenibacter (0.98%), Dialister (0.97%), Ruminococcus_2 (0.97%), Anaerostipes (0.93%), Klebsiella (0.80%), and account for 80.6% of the total sequences (**Fig. S9B**).

**SUPPLEMENTAL FIGURE LEGENDS**

**Fig. S1** Compositional and functional shift of fecal metabolites in HBV-CLD patients. Compositional shift of fecal metabolites evaluated by PCoA analysis in HCs and HBV-CLD patients (A); in cirrhotic and non-cirrhotic patients (B); in treatment-naïve and ETV-treated patients (C); KEGG enrichment analysis of differentially expressed genes between HCs and HBV-CLD patients (D). CHB, chronic hepatitis B (treatment-naive); Crrh, cirrhosis; ETV, entecavir; HBV-CLD, chronic hepatitis B virus infection-associated liver diseases; HC, healthy control; NC, non-cirrhosis.

**Fig. S2** Distinct gut microbiota signature associated with prognosis in HBV-CLD patients after fiver-year antiviral treatment. Kinetics of liver stiffness during fiver-year antiviral treatment in both regression and non-regression cohort (A); Distinct genera (B) and metabolites (C) associated with prognosis in HBV-CLD patients. Liver stiffness was summarized as median with interquartile range. The abundance of genera and metabolites was log-transformed with 0 values assigned with 1e-05. Box plots indicate median (middle line), 25th, 75th percentile (box) and maximum and minimum values (whisker). ^*^*p_fdr_*<0.05, ^**^*p_fdr_*<0.01.

**Fig. S3** Exposure of BE from treatment-naïve HBV-CLD patients and HCs on PBMCs from HCs. Treg (CD3^+^CD4^+^CD25^+^Foxp3^+^) (A), total CD8^+^ T cells (CD3^+^CD4^-^CD8^+^) (B), cytotoxic CD8^+^ T cells (CD3^+^CD4^-^CD8^+^CD45RO^-^CCR7^-^) (C), and NK cells (CD3^-^CD56^+^) (D). In response to BE exposure from HCs and HBV-CLD patients. The viability of PBMCs after BE exposure with different protein concentrations (E). Box plots indicate individual values for each sample and median with interquartile range within groups. The viability of PBMCs was shown as mean with standard deviation. BE, bacterial extracts; Crrh, cirrhosis; HBV-CLD, chronic hepatitis B virus infection-associated liver diseases; HC, healthy control; NC, non-cirrhosis; Treg, regulatory T cells.

**Fig. S4** Gating strategy for targeted cell population without differentiation condition. (A) CD4^+^ T cells (CD3^+^CD4^+^CD8^-^); (B) CD8^+^ T cells (CD3^+^CD4^-^CD8^+^); (C) cytotoxic CD8^+^ T cells (CD3^+^CD4^-^CD8^+^CD45RO^-^CCR7^-^); (D) B cells (CD3^-^CD19^+^); (E) natural killer cells (CD3^-^CD56^+^); (F) myeloid dendritic cells (CD3^-^CD19^-^CD56^+^HLADR^+^); (G) monocytes (CD3^-^ CD19^-^CD14^+^).

**Fig. S5** Gating strategy for targeted cell population under differentiation condition. (A) T regulatory cells (CD3^+^CD4^+^CD25^+^Foxp3^+^); (B) T helper 17 cells, (Th17 (CD3^+^CD4^+^IL-17A^+^); (C) T helper 1 cells (CD3^+^CD4^+^IFN-γ^+^).

**Fig. S6** Subgroup analysis of α diversity in HBV-CLD patients.α diversity analysis based on antiviral treatment (A); between patients with compensated and decompensated cirrhosis (B); Correlation of Child-Pugh Score and α diversity in cirrhotic patients (partial spearman analysis) (C); Correlation of liver stiffness and α diversity in non-cirrhotic patients (partial spearman analysis) (D). Box plots indicate median (middle line), 25th, 75th percentile (box) and maximum and minimum values (whisker). Each dot represents one sample. ^**^*p_fd_*_r_<0.01, ^***^*p_fdr_*<0.001. CC, compensated cirrhosis; DC, decompensated cirrhosis.

**Fig. S7** β diversity analysis in HBV-CLD patients. β diversity analysis (Bray-Curtis distances) in HBV-CLD patients based on the presence of cirrhosis evaluated by PCoA analysis (A) and PLS-DA analysis (B). β diversity analysis (Bray-Curtis distances) in HBV-CLD patients based on antiviral treatment evaluated by PCoA analysis (C) and PLS-DA analysis (D). CHB, chronic hepatitis B (treatment-naive); Crrh, cirrhosis; ETV, entecavir; HC, healthy control; NC, non-cirrhosis.

**Fig. S8** Subgroup analysis of β diversity in HBV-CLD patients by PCA analysis (Bray-Curtis distances). β diversity analysis in compensated and decompensated liver cirrhosis patients (A); The effect of liver stiffness on compositional shift of GM community in non-cirrhotic patients (B); The effect of Child-Pugh Score on compositional shift of GM community in cirrhotic patients (C); The effect of BMI (D), age (E) and gender (F) on compositional shift of GM community in HBV-CLD patients. CC, compensated cirrhosis; DC, decompensated cirrhosis.

**Fig. S9** Relative abundance of gut microbiota in HBV-CLD patients. Percent of community abundance on phylum level (A); on genus level (B). Data are presented as mean relative abundance within groups; Crrh, cirrhosis; HBV-CLD, chronic hepatitis B virus infection-associated liver diseases; HC, healthy control; NC, non-cirrhosis.
