# Supplementary material for: Alterations in gut microbiome and metabolomics in chronic hepatitis B infection-associated liver disease and their impact on peripheral immune response
Source: Gut Microbes. 2022 Dec 15;15(1):2155018. doi: 10.1080/19490976.2022.2155018 (PMC9757487; doi:10.1080/19490976.2022.2155018)
Supplement: Supplemental Material [file KGMI_A_2155018_SM3018.zip › 11 supplementary table.docx]

| **Table S1. The genera differentially abundant between HCs and HBV-CLD patients with or without liver cirrhosis** | | | | | | |
| --- | --- | --- | --- | --- | --- | --- |
| Genera | HC-Mean(%) | NC-Mean(%) | Crrh-Mean(%) | *P_fdr_* | | |
|  |  |  |  | HC VS NC | HC VS Crrh | NC VS Crrh |
| Faecalibacterium | 10.780 | 11.500 | 20.760 | 1.000 | **0.011** | **0.029** |
| Bifidobacterium | 4.622 | 5.429 | 1.072 | 0.051 | **0.024** | 1.000 |
| Escherichia-Shigella | 5.809 | 1.608 | 2.029 | **0.001** | **0.003** | 1.000 |
| Streptococcus | 1.468 | 1.386 | 2.514 | **0.005** | 0.922 | **0.020** |
| f__Peptostreptococcaceae | 1.068 | 0.717 | 0.493 | 0.533 | **0.000** | **0.002** |
| Clostridium_sensu_stricto_1 | 1.466 | 0.295 | 0.407 | 1.000 | 0.359 | **0.045** |
| Eubacterium_hallii_group | 0.884 | 0.543 | 0.355 | 0.564 | **0.020** | 0.494 |
| Collinsella | 0.897 | 0.459 | 0.116 | 0.109 | **0.000** | 0.286 |
| Coprococcus_2 | 0.025 | 0.723 | 0.604 | 0.064 | 1.000 | 0.106 |
| Sutterella | 0.015 | 0.162 | 0.178 | **0.029** | **0.017** | 1.000 |
| Ruminococcaceae_NK4A214_group | 0.021 | 0.256 | 0.072 | **0.035** | 1.000 | 0.065 |
| Prevotella_7 | 0.062 | 0.000 | 0.254 | 1.000 | 0.313 | **0.035** |
| Turicibacter | 0.185 | 0.036 | 0.042 | 1.000 | **0.044** | **0.032** |
| p__Saccharibacteria | 0.096 | 0.025 | 0.013 | **0.003** | **0.000** | 1.000 |
| Adlercreutzia | 0.061 | 0.046 | 0.013 | 1.000 | **0.043** | **0.015** |
| Rothia | 0.054 | 0.008 | 0.014 | **0.014** | 1.000 | **0.007** |
| Atopobium | 0.009 | 0.004 | 0.053 | 1.000 | 0.078 | 0.163 |
| Lactococcus | 0.038 | 0.000 | 0.009 | **0.001** | 0.201 | 0.061 |
| o__Clostridiales | 0.002 | 0.015 | 0.012 | **0.021** | 0.406 | 0.294 |
| Solobacterium | 0.018 | 0.002 | 0.003 | **0.001** | **0.000** | 0.905 |
| Weissella | 0.018 | 0.001 | 0.004 | **0.000** | **0.001** | 1.000 |

1. Abbreviation: Crrh, cirrhosis; HBV-CLD, hepatitis B related chronic liver disease; HC, healthy control; NC, non-cirrhosis.

2. *p_fdr_* values were calculated by Dunn’s post hoc test for 2 group comparison in genera showing statistical significance by Kruskal-Wallis for 3 group comparison (HC, NC and Crrh). *p_fdr_* < 0.05 was considered statistically significant.

| **Table S2. The genera differentially abundant between HCs and HBV-CLD with or without antiviral treatment** | | | | | | |
| --- | --- | --- | --- | --- | --- | --- |
| Genera | HC-Mean(%) | CHB-Mean(%) | ETV-Mean(%) | *pfdr* | | |
|  |  |  |  | HC vs ETV | HC vs CHB | ETV vs CHB |
| Bacteroides | 14.410 | 12.270 | 26.130 | **0.006** | 1.000 | **0.000** |
| Prevotella_9 | 3.449 | 23.210 | 3.066 | 0.548 | 0.162 | **0.000** |
| Blautia | 6.841 | 2.004 | 4.019 | 0.743 | **0.001** | **0.004** |
| Bifidobacterium | 4.622 | 3.764 | 2.006 | 0.153 | **0.001** | 0.094 |
| Escherichia-Shigella | 5.809 | 2.533 | 1.511 | **0.001** | **0.007** | 1.000 |
| Lachnoclostridium | 2.662 | 1.979 | 2.244 | 1.000 | 0.294 | **0.042** |
| Ruminococcus_torques_group | 1.319 | 0.882 | 1.965 | 1.000 | 0.248 | **0.011** |
| Fusicatenibacter | 1.664 | 0.445 | 1.001 | 1.000 | 0.059 | **0.045** |
| Ruminococcus_2 | 1.907 | 0.094 | 1.066 | 1.000 | 0.138 | **0.007** |
| Anaerostipes | 1.041 | 0.157 | 1.321 | 1.000 | 0.060 | **0.002** |
| f__Peptostreptococcaceae | 1.068 | 0.318 | 0.717 | **0.030** | 0.084 | 1.000 |
| Lachnospira | 0.389 | 0.310 | 1.214 | **0.042** | 1.000 | **0.002** |
| Parasutterella | 1.076 | 0.151 | 0.649 | 0.382 | **0.031** | 0.437 |
| Dorea | 0.794 | 0.358 | 0.593 | 1.000 | **0.032** | **0.035** |
| Eubacterium_hallii_group | 0.884 | 0.227 | 0.532 | 0.403 | **0.003** | **0.055** |
| Butyricicoccus | 0.504 | 0.338 | 0.692 | 0.577 | 0.409 | **0.003** |
| f__Lachnospiraceae | 0.405 | 0.348 | 0.618 | **0.046** | 1.000 | **0.030** |
| Collinsella | 0.897 | 0.166 | 0.280 | **0.006** | **0.003** | 1.000 |
| Erysipelotrichaceae_UCG-003 | 0.804 | 0.119 | 0.192 | 0.067 | **0.003** | 0.746 |
| f__Lachnospiraceae | 0.306 | 0.173 | 0.593 | 0.277 | 0.449 | **0.001** |
| Enterobacter | 0.701 | 0.276 | 0.046 | **0.010** | 0.058 | 1.000 |
| Intestinibacter | 0.525 | 0.135 | 0.245 | 0.617 | 1.000 | **0.049** |
| Ruminococcaceae_UCG-013 | 0.409 | 0.110 | 0.293 | 1.000 | **0.007** | **0.000** |
| Eubacterium_ventriosum_group | 0.139 | 0.087 | 0.264 | 1.000 | 0.176 | **0.010** |
| Lachnospiraceae_ND3007_group | 0.118 | 0.062 | 0.245 | **0.011** | 1.000 | **0.006** |
| Sutterella | 0.015 | 0.127 | 0.198 | **0.008** | 0.087 | 1.000 |
| Tyzzerella_3 | 0.077 | 0.017 | 0.160 | 0.338 | 0.474 | **0.001** |
| Lachnospiraceae_UCG-010 | 0.037 | 0.075 | 0.139 | **0.020** | 1.000 | 0.203 |
| f__Ruminococcaceae | 0.057 | 0.074 | 0.120 | **0.036** | 0.985 | 0.342 |
| Catenibacterium | 0.000 | 0.063 | 0.100 | 1.000 | 0.080 | 0.062 |
| p__Saccharibacteria | 0.096 | 0.025 | 0.013 | **0.000** | **0.003** | 1.000 |
| Ruminiclostridium_9 | 0.011 | 0.044 | 0.068 | **0.015** | 1.000 | **0.047** |
| Eubacterium_xylanophilum_group | 0.004 | 0.005 | 0.059 | 0.066 | 1.000 | **0.022** |
| Lachnospiraceae_UCG-003 | 0.002 | 0.006 | 0.047 | 0.204 | 1.000 | 0.095 |
| Lactococcus | 0.038 | 0.009 | 0.004 | **0.014** | 0.156 | 1.000 |
| Solobacterium | 0.018 | 0.004 | 0.002 | **0.000** | **0.013** | 0.944 |
| Weissella | 0.018 | 0.001 | 0.004 | **0.001** | **0.000** | 1.000 |

1. Abbreviation: CHB, treatment naïve chronic hepatitis B; ETV, entecavir; HBV-CLD, hepatitis B related chronic liver disease; HC, healthy control.

2. *p_fdr_* values were calculated by Dunn’s post hoc test for 2 group comparison in genera showing statistical significance by Kruskal-Wallis for 3 group comparison (HC, CHB and ETV). *p_fdr_* < 0.05 was considered statistically significant.

| **Table S3. Distinct metabolites in the HBV-CLD patients compared to the HCs** | | | | |
| --- | --- | --- | --- | --- |
| Metabolite | VIP | FC | *p* | *p_fdr_* |
| APC | 4.316 | 5.658 | 0.000 | 0.000 |
| 3-beta-Hydroxy-5-cholestenoic acid | 3.628 | 0.351 | 0.000 | 0.001 |
| O-Desmethyltramadol | 3.473 | 0.345 | 0.000 | 0.003 |
| 2-Linoleoylglycerophosphocholine | 3.445 | 0.466 | 0.000 | 0.003 |
| Esculentoside E | 3.389 | 2.062 | 0.000 | 0.001 |
| Asparaginyl-Glutamine | 3.336 | 0.464 | 0.000 | 0.000 |
| Presqualene diphosphate | 3.241 | 0.602 | 0.000 | 0.000 |
| 6-amino-5[N-methylformylamino]-1-methyluracil | 3.190 | 0.494 | 0.000 | 0.007 |
| Cubebinin | 3.172 | 2.332 | 0.000 | 0.000 |
| Goshonoside F3 | 3.093 | 0.523 | 0.000 | 0.006 |
| PE(15:0/18:4(6Z,9Z,12Z,15Z)) | 3.071 | 0.628 | 0.000 | 0.000 |
| Glycylalanylprolylmethionylphenylalanylvalinamide | 3.025 | 2.126 | 0.000 | 0.002 |
| Ile-Asp-OH | 2.937 | 2.165 | 0.000 | 0.002 |
| Bestatin (hydrochloride) | 2.929 | 0.596 | 0.000 | 0.008 |
| 5-Acetylamino-6-amino-3-methyluracil | 2.913 | 0.538 | 0.001 | 0.011 |
| Ritalinic acid | 2.895 | 0.448 | 0.000 | 0.003 |
| Cycloartomunoxanthone | 2.837 | 0.442 | 0.001 | 0.008 |
| 2,3-Dihydro-2-S-glutathionyl-3-hydroxy bromobenzene | 2.785 | 0.643 | 0.000 | 0.004 |
| Murrayacine | 2.782 | 0.662 | 0.000 | 0.002 |
| (-)-Stercobilin | 2.636 | 0.808 | 0.000 | 0.004 |
| 5-Methyl-2,5-di-1-pyrrolidinyl-2-cyclopenten-1-one | 2.624 | 0.660 | 0.000 | 0.003 |
| Formononetin | 2.593 | 1.705 | 0.000 | 0.007 |
| Austalide H | 2.582 | 0.702 | 0.000 | 0.002 |
| Steviobioside | 2.574 | 0.736 | 0.000 | 0.004 |
| N-(6-aminohexanoyl)-6-aminohexanoic acid | 2.573 | 0.649 | 0.000 | 0.007 |
| (S)-3-Hydroxyisobutyric acid | 2.564 | 0.718 | 0.000 | 0.003 |
| Simonin IV | 2.561 | 0.803 | 0.000 | 0.001 |
| Sulfolithocholylglycine | 2.538 | 0.655 | 0.001 | 0.014 |
| Moracin P | 2.531 | 1.640 | 0.000 | 0.006 |
| 5-(2-Hydroxyethyl)-4-methylthiazole acetate | 2.522 | 0.601 | 0.000 | 0.005 |
| (3beta,5alpha,6alpha,7alpha,22E,24R)-5,6-Epoxyergosta-8,14,22-triene-3,7-diol | 2.514 | 0.702 | 0.000 | 0.005 |
| Capsianoside IV | 2.511 | 0.652 | 0.001 | 0.016 |
| Beraprost | 2.510 | 0.533 | 0.001 | 0.013 |
| Minocycline | 2.498 | 1.713 | 0.000 | 0.002 |
| Valyl-Hydroxyproline | 2.474 | 1.637 | 0.001 | 0.015 |
| Neoconvallatoxoloside | 2.462 | 0.764 | 0.000 | 0.004 |
| 4-Hydroxyphenytoin glucuronide | 2.461 | 1.567 | 0.000 | 0.002 |
| Permetin A | 2.437 | 0.787 | 0.000 | 0.003 |
| 9,10-DiHODE | 2.427 | 0.782 | 0.000 | 0.003 |
| Lacto-N-triaose | 2.426 | 1.498 | 0.000 | 0.007 |
| Norrubrofusarin 6-beta-gentiobioside | 2.413 | 1.439 | 0.001 | 0.013 |
| S-(11-hydroxy-9-deoxy-delta12-PGD2)-glutathione | 2.409 | 0.678 | 0.001 | 0.011 |
| 7-Hydroxy-2-methyl-4-oxo-4H-1-benzopyran-5-carboxylic acid 7-glucoside | 2.399 | 0.694 | 0.000 | 0.003 |
| MG(0:0/16:0/0:0) | 2.388 | 0.667 | 0.003 | 0.022 |
| Ginsenoyne C | 2.361 | 0.769 | 0.000 | 0.005 |
| CL(8:0/8:0/12:0/18:2(9Z,11Z)) | 2.360 | 0.746 | 0.000 | 0.002 |
| Aflatoxin M2 | 2.339 | 0.634 | 0.001 | 0.010 |
| Cis-3-(1-Carboxy-ethyl)-3,5-cyclo-hexadiene-1,2-diol | 2.337 | 0.791 | 0.000 | 0.002 |
| Inundatine | 2.325 | 0.649 | 0.001 | 0.009 |
| 2-Propenyl heptanoate | 2.324 | 1.294 | 0.000 | 0.005 |
| 2-Isopropyl-3-oxosuccinate | 2.322 | 0.610 | 0.004 | 0.029 |
| 14-HDoHE | 2.316 | 0.752 | 0.000 | 0.003 |
| Nonivamide | 2.310 | 0.702 | 0.000 | 0.004 |
| Methoxybrassenin B | 2.309 | 0.728 | 0.000 | 0.002 |
| Geniposidic Acid | 2.302 | 0.684 | 0.001 | 0.008 |
| Cynaroside A | 2.291 | 1.865 | 0.001 | 0.013 |
| Digitoxigenin | 2.288 | 0.733 | 0.001 | 0.009 |
| Alanyl-Methionine | 2.270 | 1.604 | 0.003 | 0.024 |
| DG(16:1(9Z)/20:3(5Z,8Z,11Z)/0:0) | 2.264 | 0.680 | 0.001 | 0.014 |
| 1,3,7-Trimethyluric acid | 2.263 | 0.703 | 0.005 | 0.032 |
| Cytidine | 2.243 | 0.703 | 0.001 | 0.009 |
| Taurochenodeoxycholate-3-sulfate | 2.240 | 1.295 | 0.000 | 0.007 |
| Hydroxystrobilurin D | 2.220 | 1.392 | 0.000 | 0.004 |
| Erinacine A | 2.218 | 0.622 | 0.005 | 0.032 |
| (R)-Pantothenic acid 4'-O-b-D-glucoside | 2.216 | 1.741 | 0.001 | 0.014 |
| Mesuaxanthone B | 2.202 | 0.743 | 0.001 | 0.014 |
| (S)-Cajaflavanone | 2.200 | 1.451 | 0.002 | 0.018 |
| 13-Nor-6-eremophilene-8,11-dione | 2.198 | 0.666 | 0.000 | 0.007 |
| 4-(4-Hydroxyphenyl)-2-butanone | 2.195 | 0.636 | 0.006 | 0.036 |
| 24-Acetyl- 25-cinnamoylvulgaroside | 2.193 | 1.510 | 0.004 | 0.031 |
| Butyl 2-aminobenzoate | 2.190 | 0.718 | 0.000 | 0.004 |
| LysoPA(a-13:0/0:0) | 2.187 | 1.308 | 0.000 | 0.002 |
| Musabalbisiane C | 2.186 | 1.425 | 0.002 | 0.017 |
| Eriojaposide A | 2.181 | 1.299 | 0.000 | 0.002 |
| Chondroitin 4-sulfate | 2.181 | 1.516 | 0.008 | 0.044 |
| Cis-Miyabenol C | 2.172 | 0.728 | 0.001 | 0.015 |
| Urolithin A | 2.165 | 0.734 | 0.009 | 0.047 |
| 5-Methoxycanthin-6-one | 2.153 | 1.518 | 0.003 | 0.023 |
| A-L-threo-4-Hex-4-enopyranuronosyl-D-galacturonic acid | 2.153 | 1.221 | 0.000 | 0.004 |
| Neotame | 2.148 | 1.280 | 0.000 | 0.006 |
| 13-Oxocryptopine | 2.142 | 1.983 | 0.009 | 0.050 |
| (3b,6b,8b,12a)-8,12-Epoxy-7(11)-eremophilene-6-angeloyloxy-8,12-dimethoxy-3-ol | 2.130 | 1.341 | 0.000 | 0.004 |
| N-gamma-Glutamyl-S-allylcysteine | 2.126 | 0.643 | 0.008 | 0.045 |
| 17-phenoxy trinor Prostaglandin F2alpha | 2.120 | 0.770 | 0.000 | 0.006 |
| Isobutyryl-L-carnitine | 2.118 | 0.710 | 0.000 | 0.007 |
| Alpha-Phenylcyclohexylglycolic acid | 2.117 | 0.483 | 0.004 | 0.030 |
| N-Acetylmuramate | 2.113 | 0.770 | 0.000 | 0.005 |
| Protoporphyrinogen IX | 2.101 | 0.772 | 0.002 | 0.019 |
| Histidinyl-Threonine | 2.090 | 0.624 | 0.001 | 0.013 |
| Fumigaclavine A | 2.086 | 0.713 | 0.002 | 0.020 |
| Stachyoside A | 2.085 | 1.334 | 0.002 | 0.018 |
| 7-Ethyl-2,3,6,7-tetrahydrocyclopent[b]azepin-8(1H)-one | 2.076 | 0.757 | 0.000 | 0.003 |
| 4-Hydroxyretinoic acid | 2.075 | 0.755 | 0.001 | 0.014 |
| 4'-Hydroxycarvedilol | 2.061 | 1.611 | 0.006 | 0.036 |
| Diethylpropion (metabolite XI Glucuronide) | 2.057 | 0.756 | 0.001 | 0.011 |
| Physapubescin | 2.046 | 0.738 | 0.001 | 0.015 |
| 7a,12a-Dihydroxy-3-oxo-4-cholenoic acid | 2.038 | 1.274 | 0.000 | 0.002 |
| Postin | 2.037 | 0.649 | 0.008 | 0.045 |
| Cer(d18:0/15:0) | 2.035 | 0.818 | 0.000 | 0.006 |
| 7-Hydroxyenterolactone | 2.026 | 0.803 | 0.001 | 0.011 |
| Baptifoline | 2.026 | 1.395 | 0.001 | 0.014 |
| (2S,4S)-Monatin | 2.021 | 1.433 | 0.002 | 0.016 |
| 1,7-Dimethyluric acid | 2.018 | 0.738 | 0.007 | 0.043 |
| Cis-3-Hexenyl b-primeveroside | 2.015 | 1.355 | 0.000 | 0.004 |
| Cytochalasin Ppho | 2.008 | 0.727 | 0.001 | 0.009 |
| 3-(3-Hydroxyphenyl)-3-hydroxypropanoic acid | 2.005 | 0.701 | 0.001 | 0.011 |
| Janthitrem B | 2.000 | 1.329 | 0.001 | 0.009 |
| Isopentyl gentiobioside | 1.997 | 0.788 | 0.000 | 0.006 |
| 13,14-dehydro-15-cyclohexyl Carbaprostacyclin | 1.995 | 0.769 | 0.000 | 0.005 |
| Corchorusoside B | 1.994 | 0.767 | 0.003 | 0.026 |
| Hydroxyprolyl-Serine | 1.992 | 1.663 | 0.002 | 0.020 |
| Ferulic acid | 1.989 | 1.230 | 0.000 | 0.004 |
| 3-(3,5-dihydroxyphenyl)-1-propanoic acid sulphate | 1.962 | 1.392 | 0.006 | 0.038 |
| Folinic acid | 1.933 | 1.309 | 0.005 | 0.034 |
| Hordatine A | 1.933 | 1.300 | 0.000 | 0.005 |
| Furaneol 4-(6-malonylglucoside) | 1.932 | 1.458 | 0.002 | 0.018 |
| 1-O-[(2e)-3-(2-hydroxyphenyl)-1-oxo-2-propen-1-yl]-beta-D-Glucopyranose | 1.930 | 0.729 | 0.003 | 0.027 |
| 1-(3-Methoxy-4-hydroxy)-phenyl-6,7-dihydroxy-isochroman | 1.922 | 0.710 | 0.003 | 0.022 |
| 5b-Cyprinol sulfate | 1.920 | 1.314 | 0.003 | 0.023 |
| Caffeoyl tyrosine | 1.919 | 1.249 | 0.000 | 0.001 |
| Ganoderic acid alpha | 1.918 | 1.208 | 0.000 | 0.006 |
| PE(15:0/20:4(5Z,8Z,11Z,14Z)) | 1.917 | 0.829 | 0.000 | 0.001 |
| Theophylline | 1.914 | 0.778 | 0.002 | 0.018 |
| 6-Hydroxyenterolactone | 1.905 | 0.797 | 0.002 | 0.016 |
| Thromboxane B3 | 1.902 | 1.342 | 0.000 | 0.006 |
| Melatonin radical | 1.890 | 0.627 | 0.007 | 0.041 |
| Gamma-Tocotrienol | 1.885 | 0.804 | 0.004 | 0.027 |
| 5'-Hydroxy-3',4',7,8-tetramethoxyflavan | 1.885 | 0.746 | 0.001 | 0.014 |
| Tryptophol [xylosyl-(1->6)-glucoside] | 1.880 | 1.301 | 0.000 | 0.007 |
| 7-Hydroxy-2,5-dimethyl-4H-1-benzopyran-4-one | 1.879 | 0.776 | 0.001 | 0.009 |
| 1-(11Z-docosenoyl)-glycero-3-phosphate | 1.874 | 0.802 | 0.000 | 0.008 |
| 6-hydroxyoct-6-enoylglycine | 1.873 | 0.777 | 0.005 | 0.032 |
| 3-Hydroxytetradecanedioic acid | 1.872 | 0.737 | 0.005 | 0.032 |
| 1-heptadecanoyl-glycero-3-phosphate | 1.869 | 1.301 | 0.002 | 0.017 |
| Isorhamnetin | 1.867 | 1.236 | 0.006 | 0.037 |
| Genistein | 1.862 | 1.239 | 0.009 | 0.050 |
| 4-Carboxyphenylglycine | 1.858 | 0.810 | 0.000 | 0.004 |
| Valinopine | 1.855 | 0.826 | 0.001 | 0.008 |
| 1-(9Z-tetradecenoyl)-glycero-3-phosphate | 1.853 | 1.290 | 0.000 | 0.007 |
| Tranylcypromine glucuronide | 1.853 | 0.723 | 0.006 | 0.040 |
| Cer(d14:1/18:0) | 1.839 | 0.804 | 0.000 | 0.007 |
| Trigonelline | 1.829 | 0.752 | 0.000 | 0.008 |
| 3,4,5-trihydroxy-6-(3-methyl-2-oxo-4-phenylbutoxy)oxane-2-carboxylic acid | 1.829 | 0.784 | 0.001 | 0.015 |
| Tryptophanamide | 1.825 | 0.797 | 0.001 | 0.010 |
| 7,8-Dihydrovomifoliol 9-[rhamnosyl-(1->6)-glucoside] | 1.823 | 1.278 | 0.001 | 0.009 |
| Histidinyl-Gamma-glutamate | 1.822 | 0.757 | 0.006 | 0.036 |
| (4E,8E,10E-d18:3)sphingosine | 1.811 | 0.790 | 0.000 | 0.002 |
| Capsicum annuum Fluorescent chlorophyll catabolite | 1.804 | 1.288 | 0.008 | 0.045 |
| Cotinine | 1.798 | 0.759 | 0.001 | 0.015 |
| Ginsenoyne B | 1.797 | 0.813 | 0.003 | 0.023 |
| Phenylglucuronide | 1.791 | 0.683 | 0.008 | 0.047 |
| 4-(Methylnitrosamino)-1-(3-pyridyl)-1-butanone | 1.790 | 0.803 | 0.001 | 0.013 |
| Cytosine | 1.789 | 1.394 | 0.002 | 0.017 |
| 5a-Cholestane-3a,7a,12a,25-tetrol | 1.777 | 0.817 | 0.000 | 0.004 |
| 6b-Hydroxy-8a-methoxy-7(11)-eremophilen-12,8-olide | 1.775 | 0.819 | 0.000 | 0.007 |
| Ustiloxin C | 1.768 | 0.779 | 0.002 | 0.020 |
| Benzoquinoneacetic acid | 1.766 | 0.767 | 0.001 | 0.010 |
| 3',4,4'-Trihydroxypulvinone | 1.765 | 1.400 | 0.005 | 0.035 |
| (3beta,5alpha,9alpha,22E,24R)-5,9-Epidioxy-3-hydroxyergosta-7,22-dien-6-one | 1.763 | 0.820 | 0.003 | 0.027 |
| Cer(d18:0/12:0) | 1.762 | 0.808 | 0.001 | 0.011 |
| Dimethylbenzyl carbinyl hexanoate | 1.757 | 1.282 | 0.002 | 0.021 |
| Methionyl-Hydroxyproline | 1.757 | 0.811 | 0.001 | 0.014 |
| 2-Methylerythritol | 1.753 | 0.818 | 0.001 | 0.009 |
| (9Z,11E,13E,15Z)-4-Oxo-9,11,13,15-octadecatetraenoic acid | 1.748 | 0.758 | 0.007 | 0.041 |
| 2-Pyridyl hydroxymethane sulfonic acid | 1.744 | 0.733 | 0.006 | 0.037 |
| L-Arogenate | 1.730 | 0.826 | 0.000 | 0.003 |
| 11-bromo-undecanoic acid | 1.721 | 0.808 | 0.002 | 0.017 |
| Secoeremopetasitolide A | 1.718 | 1.249 | 0.002 | 0.021 |
| 3,5-Dimethoxy-2,7-phenanthrenediol | 1.717 | 0.791 | 0.005 | 0.032 |
| 7-Hydroxy-3,7-dimethyloctanal | 1.716 | 0.792 | 0.001 | 0.011 |
| ROSAVIN | 1.716 | 1.203 | 0.003 | 0.022 |
| ANHYDROBRAZILIC ACID | 1.703 | 0.793 | 0.001 | 0.011 |
| Taurochenodeoxycholate-7-sulfate | 1.702 | 1.262 | 0.008 | 0.046 |
| (R)-Juziphine | 1.701 | 1.246 | 0.001 | 0.016 |
| Allyl undecylenate | 1.697 | 0.781 | 0.007 | 0.040 |
| calcitriol | 1.688 | 0.829 | 0.002 | 0.020 |
| 17-phenyl trinor PGF2alpha isopropyl ester | 1.686 | 0.783 | 0.008 | 0.047 |
| 2-Hydroxy-3-methyl-4H-pyran-4-one O-(6E-cinnamoyl-b-D-glucoside) | 1.682 | 1.223 | 0.001 | 0.016 |
| LysoPA(0:0/18:1(9Z)) | 1.681 | 1.309 | 0.008 | 0.045 |
| Domoic acid | 1.664 | 1.377 | 0.009 | 0.048 |
| Dihydrovaltrate | 1.658 | 1.295 | 0.005 | 0.034 |
| 8-Hydroxycarvedilol | 1.656 | 0.825 | 0.000 | 0.006 |
| Cycloartocarpesin | 1.637 | 0.775 | 0.007 | 0.040 |
| Elenaic acid | 1.633 | 0.795 | 0.005 | 0.036 |
| Aeglin | 1.629 | 1.307 | 0.007 | 0.041 |
| Oryzalide A | 1.620 | 1.203 | 0.001 | 0.012 |
| O-Phosphotyrosine | 1.615 | 0.794 | 0.006 | 0.036 |
| 2-{[hydroxy(1-oxo-1H-isochromen-3-yl)methylidene]amino}acetic acid | 1.614 | 1.324 | 0.005 | 0.035 |
| 2,4-Di-tert-butylphenol | 1.612 | 0.799 | 0.005 | 0.032 |
| 12-Hydroxynevirapine glucuronide | 1.585 | 1.243 | 0.003 | 0.026 |
| Ethyl glucuronide | 1.581 | 1.301 | 0.006 | 0.036 |
| Alectrol | 1.571 | 0.829 | 0.004 | 0.029 |
| Myricanol 5-beta-sophoroside | 1.566 | 1.253 | 0.008 | 0.046 |
| 1,2-Propanediol, 3-[[2-(2-methoxyphenoxy)ethyl]amino] | 1.565 | 0.792 | 0.003 | 0.023 |
| 5S,6S-epoxy-15R-hydroxy-ETE | 1.555 | 1.221 | 0.005 | 0.031 |
| Pelargonic acid | 1.549 | 1.242 | 0.003 | 0.026 |
| ALANYL-dl-LEUCINE | 1.542 | 0.821 | 0.001 | 0.011 |
| L-tryptophan | 1.538 | 1.201 | 0.002 | 0.021 |
| Allobarbital | 1.534 | 1.236 | 0.008 | 0.045 |
| 11beta,17,21-Trihydroxy-2alpha-methylpregn-4-ene-3,20-dione 21-acetate | 1.521 | 0.826 | 0.004 | 0.028 |
| 3-Methylindolepyruvate | 1.515 | 0.825 | 0.005 | 0.035 |
| 2-(3-Hydroxyphenyl)ethanol 1'-glucoside | 1.467 | 1.237 | 0.007 | 0.043 |
| 2-hydroxyundecanoic acid | 1.423 | 0.828 | 0.006 | 0.037 |
| 9-Oxoasimicinone | 1.407 | 0.828 | 0.004 | 0.030 |
| 3-Hydroxy-2H-pyran-2-one | 1.333 | 1.202 | 0.008 | 0.047 |
| 8-(Methylthio)octanenitrile | 1.284 | 0.816 | 0.007 | 0.042 |

1. Abbreviation: FC, fold change; HBV-CLD, hepatitis B related chronic liver disease; HC, healthy control; VIP, variable importance in the projection.

2. *p_fdr_* values were calculated by Mann–Whitney U test. *p_fdr_* < 0.05 was considered statistically significant.

| **Table S4. Comparison of immune cell populations in treatment naïve HBV-CLD patients and healthy controls** | | | | | | | |
| --- | --- | --- | --- | --- | --- | --- | --- |
| Variable | HC | NC | Crrh | Global test | Pairwise test | | |
|  |  |  |  | HC VS NC VS Crrh | HC VS NC | HC VS Crrh | NC VS Crrh |
| Tregs | 0.046 (±0.021) | 0.033 (±0.016) | 0.027 (±0.014) | 0.041 | 0.173 | 0.036 | 0.720 |
| Th17 | 0.042 (±0.016) | 0.072 (±0.021) | 0.060 (±0.017) | 0.005 | 0.003 | 0.091 | 0.274 |
| Th1 | 0.127 (±0.026) | 0.101 (±0.026) | 0.086 (±0.026) | 0.003 | 0.065 | 0.002 | 0.331 |
| CD4^+^ T cells | 0.529 (±0.121) | 0.490 (±0.141) | 0.493 (±0.136) | 0.714 | 0.736 | 0.778 | 0.998 |
| CD8^+^ T cells | 0.315 (±0.103) | 0.248 (±0.127) | 0.240 (±0.139) | 0.594 | 0.358 | 0.290 | 0.984 |
| Cytotoxic CD8+ T cells | 0.825 (±0.105) | 0.879 (±0.082) | 0.885 (±0.091) | 0.198 | 0.293 | 0.233 | 0.984 |
| Monocytes | 0.088 (±0.067) | 0.025 (±0.020) | 0.010 (±0.198) | 0.000 | 0.001 | 0.001 | 0.984 |
| DC | 1.685 (±2.442) | 1.876 (±2.247) | 1.732 (±2.371) | 0.977 | 0.977 | 0.999 | 0.986 |
| B cells | 0.280 (±0.175) | 0.284 (±0.153) | 0.274 (±0.157) | 0.988 | 0.998 | 0.995 | 0.987 |
| NK cells | 0.100 (±0.076) | 0.064 (±0.038) | 0.066 (±0.033) | 0.178 | 0.214 | 0.264 | 0.995 |

1. Abbreviation: Crrh, cirrhosis; DC, dendritic cells; HC, healthy control; NC, non-cirrhosis; NK, natural killer; SE, standard error; Th17, T helper 17 cells; Th1, T helper 1 cells; Tregs, T regular cells.

2. Data is shown as mean ± standard error.

3. *p* values were calculated via two tailed t-test for two group comparison or one-way ANOVA for 3 group comparison with Tukey’s post hoc test for 2 group comparisons (HC, NC and Crrh). *p* < 0.05 was considered statistically significant.

**Table S5. The alpha‐diversity indexes of the gut microbiota in the different groups**

| Indexes | HC | ETV | CHB | CC | DC | Global test | Pairwise test | | | CC vs DC |
| --- | --- | --- | --- | --- | --- | --- | --- | --- | --- | --- |
|  |  |  |  |  |  | HC vs CHB vs ETV | HC vs CHB | HC vs ETV | CHB vs ETV |  |
| Shannon | 3.31 (2.55, 3.42) | 3.18 (3.08, 3.45) | 2.73 (2.46, 2.83) | 3.20 (3.14, 3.43) | 2.94 (2.70, 3.16) | **0.004** | **0.004** | 0.349 | **0.000** | **0.000** |
| Simpson | 0.07 (0.06, 0.18) | 0.09 (0.07, 0.12) | 0.17 (0.14, 0.21) | 0.09 (0.08, 0.11) | 0.14 (0.10, 0.17) | **0.000** | **0.000** | 0.156 | **0.000** | **0.002** |
| Shannoneven | 0.63 (0.54, 0.67) | 0.62 (0.58, 0.66) | 0.53 (0.47, 0.56) | 0.64 (0.61, 0.65) | 0.56 (0.54, 0.63) | **0.000** | **0.001** | 0.419 | **0.000** | **0.003** |
| Simpsoneven | 0.07 (0.03, 0.10) | 0.06 (0.05, 0.09) | 0.04 (0.03, 0.05) | 0.06 (0.05, 0.09) | 0.05 (0.03, 0.07) | **0.001** | **0.003** | 0.715 | **0.003** | 0.092 |

1. Abbreviation: CC, compensated cirrhosis; CHB, chronic hepatitis B; Crrh, cirrhosis; DC, decompensated cirrhosis; ETV, entecavir; HC, healthy control; HBV-CLD, chronic hepatitis B virus infection-associated liver diseases; NC, non-cirrhosis; No, number; SD, standard deviation.

2. Quantitative results are expressed as median with first and third quartiles into brackets.

3. *p* values were calculated by Mann–Whitney U test for 2 group comparison (CC vs DC) and Kruskal–Wallis for 3 group comparison with Dunn’s post hoc test for 2 group comparison (HC, CHB and ETV); *p* < 0.05 was considered statistically significant.
