# Supplementary figures and images for: Alterations in gut microbiome and metabolomics in chronic hepatitis B infection-associated liver disease and their impact on peripheral immune response
Source: Gut Microbes. 2022 Dec 15;15(1):2155018. doi: 10.1080/19490976.2022.2155018 (PMC9757487; doi:10.1080/19490976.2022.2155018)

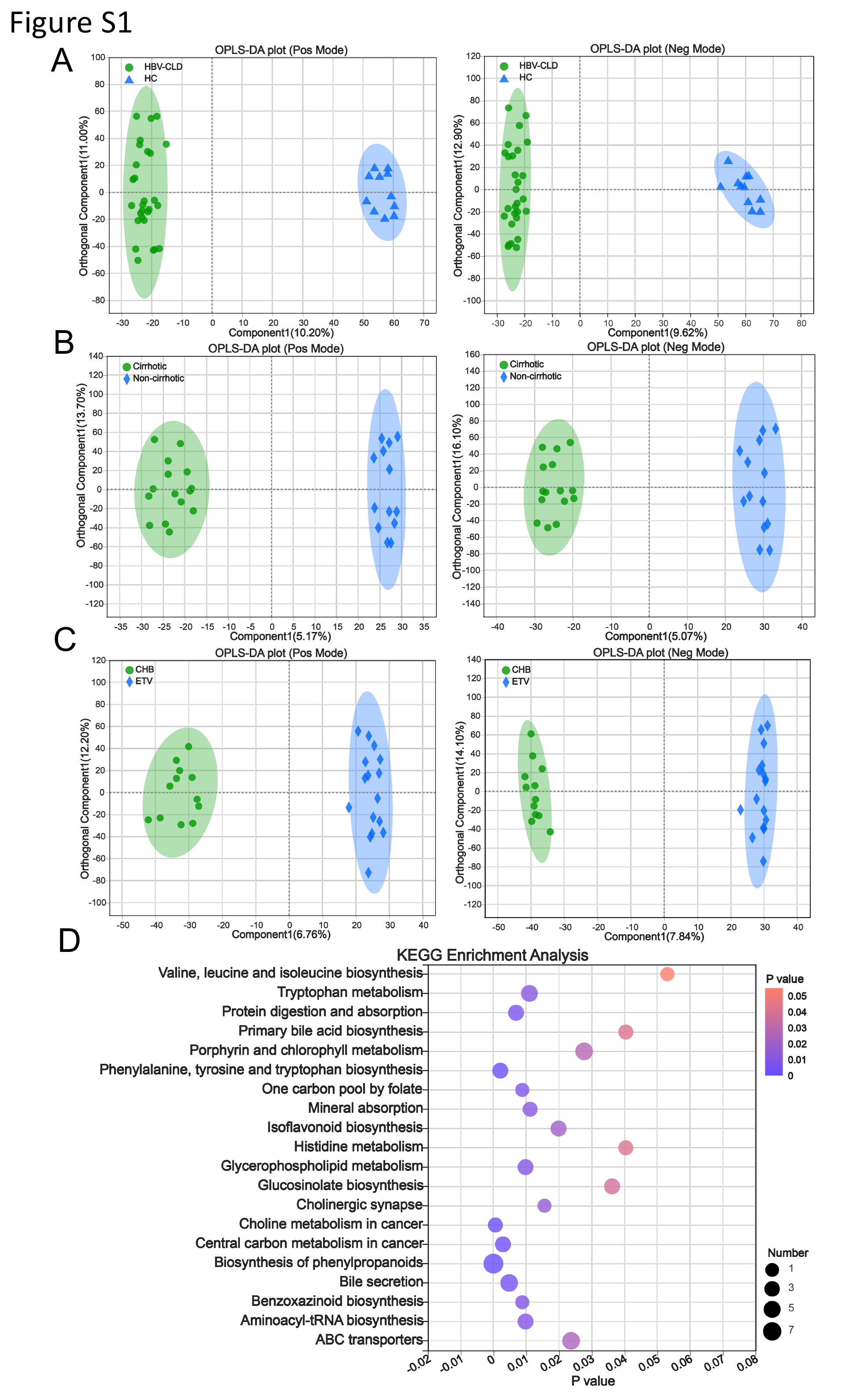

Supplement: Supplemental Material [file KGMI_A_2155018_SM3018.zip › 12 figure S1.png]

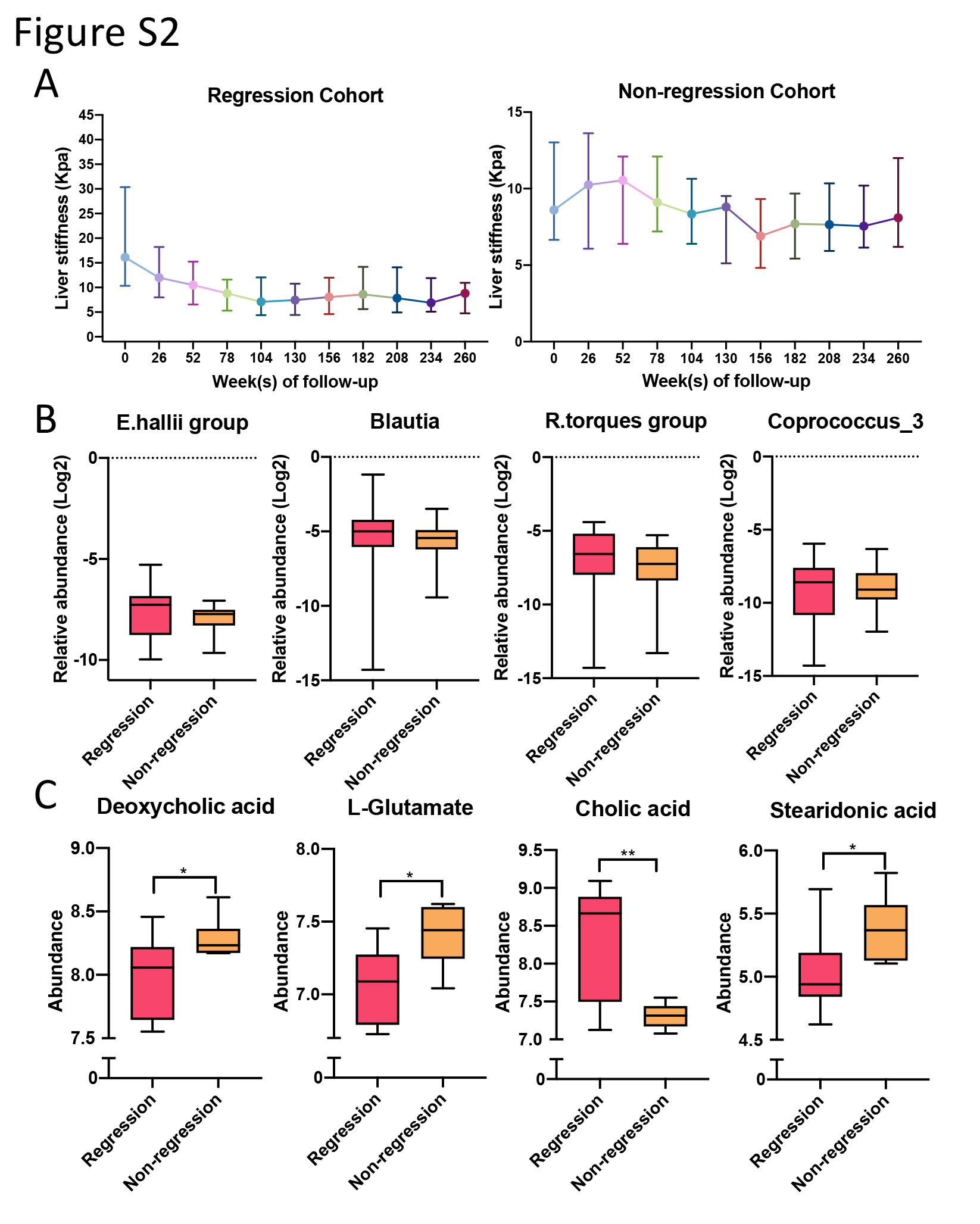

Supplement: Supplemental Material [file KGMI_A_2155018_SM3018.zip › 13 figure S2.png]

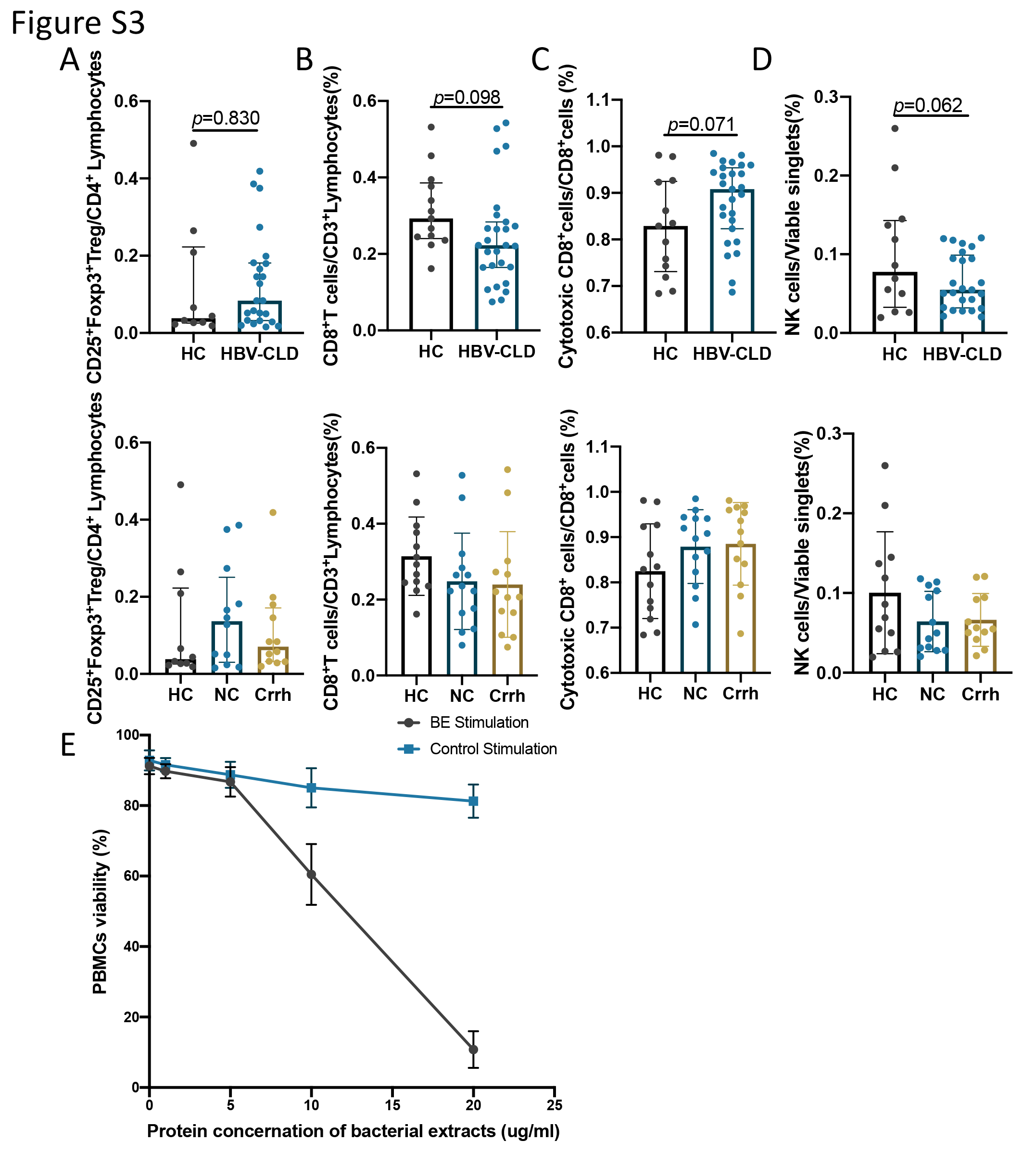

Supplement: Supplemental Material [file KGMI_A_2155018_SM3018.zip › 14 figure S3.png]

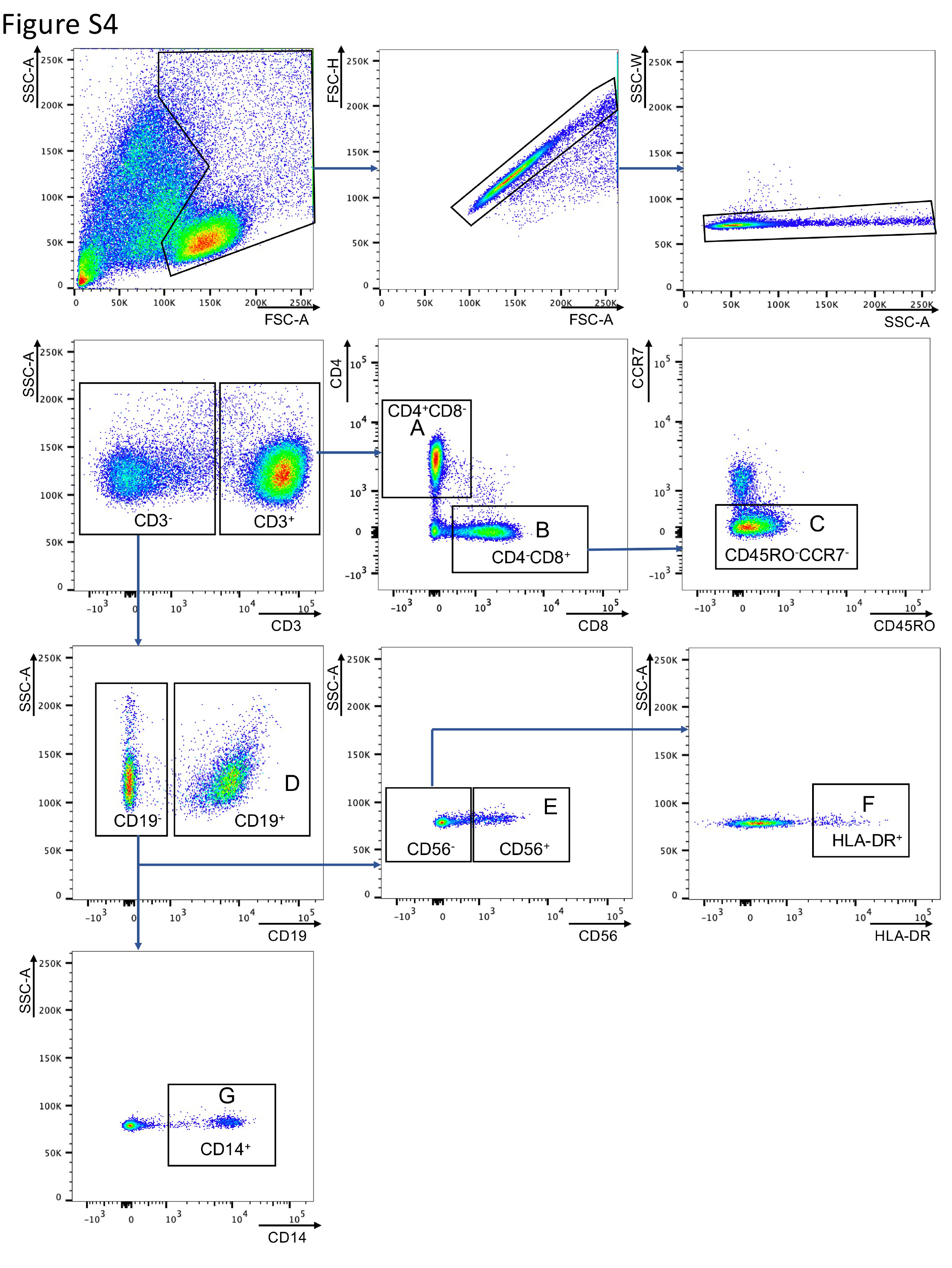

Supplement: Supplemental Material [file KGMI_A_2155018_SM3018.zip › 15 figure S4.png]

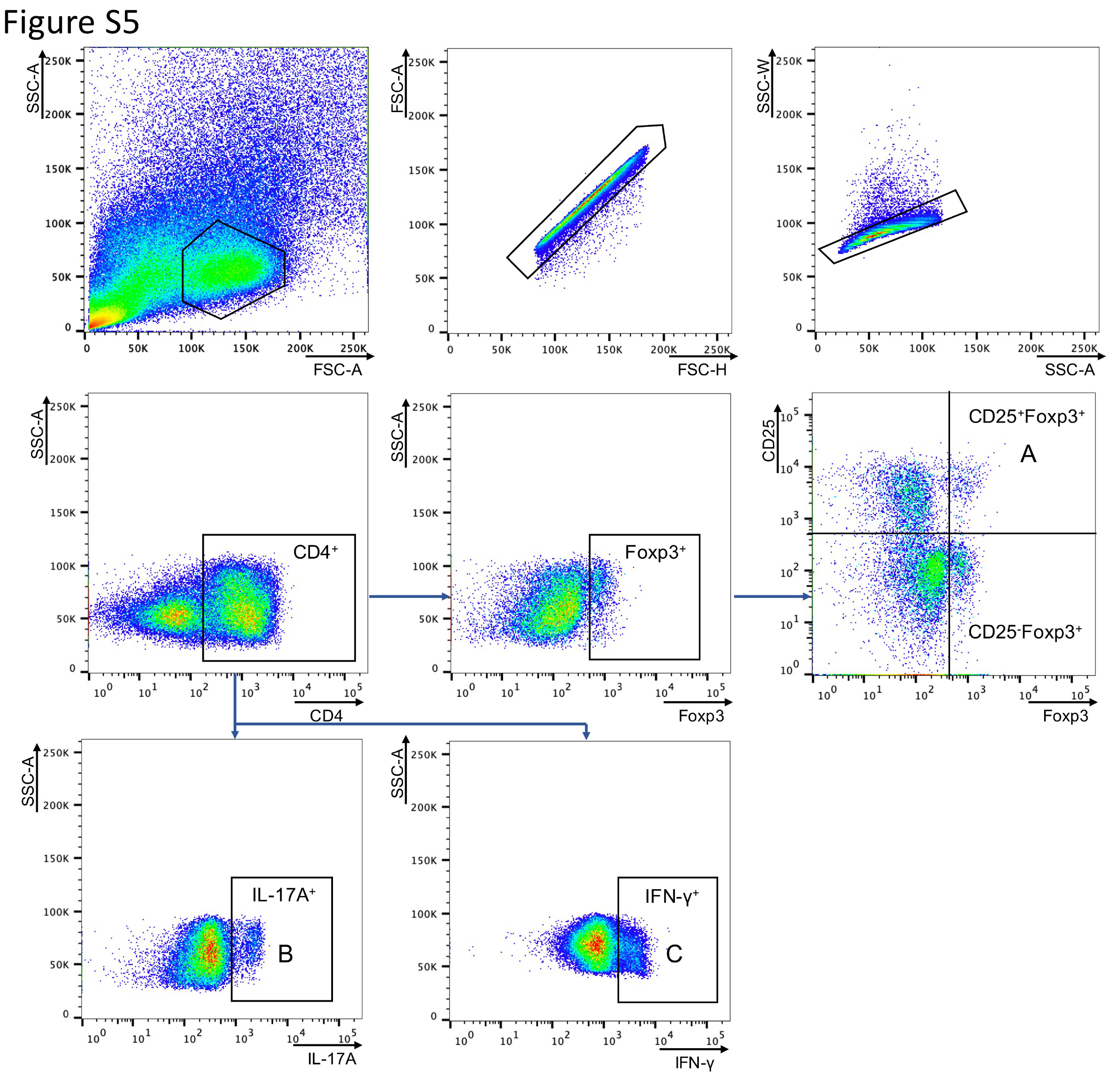

Supplement: Supplemental Material [file KGMI_A_2155018_SM3018.zip › 16 figure S5.png]

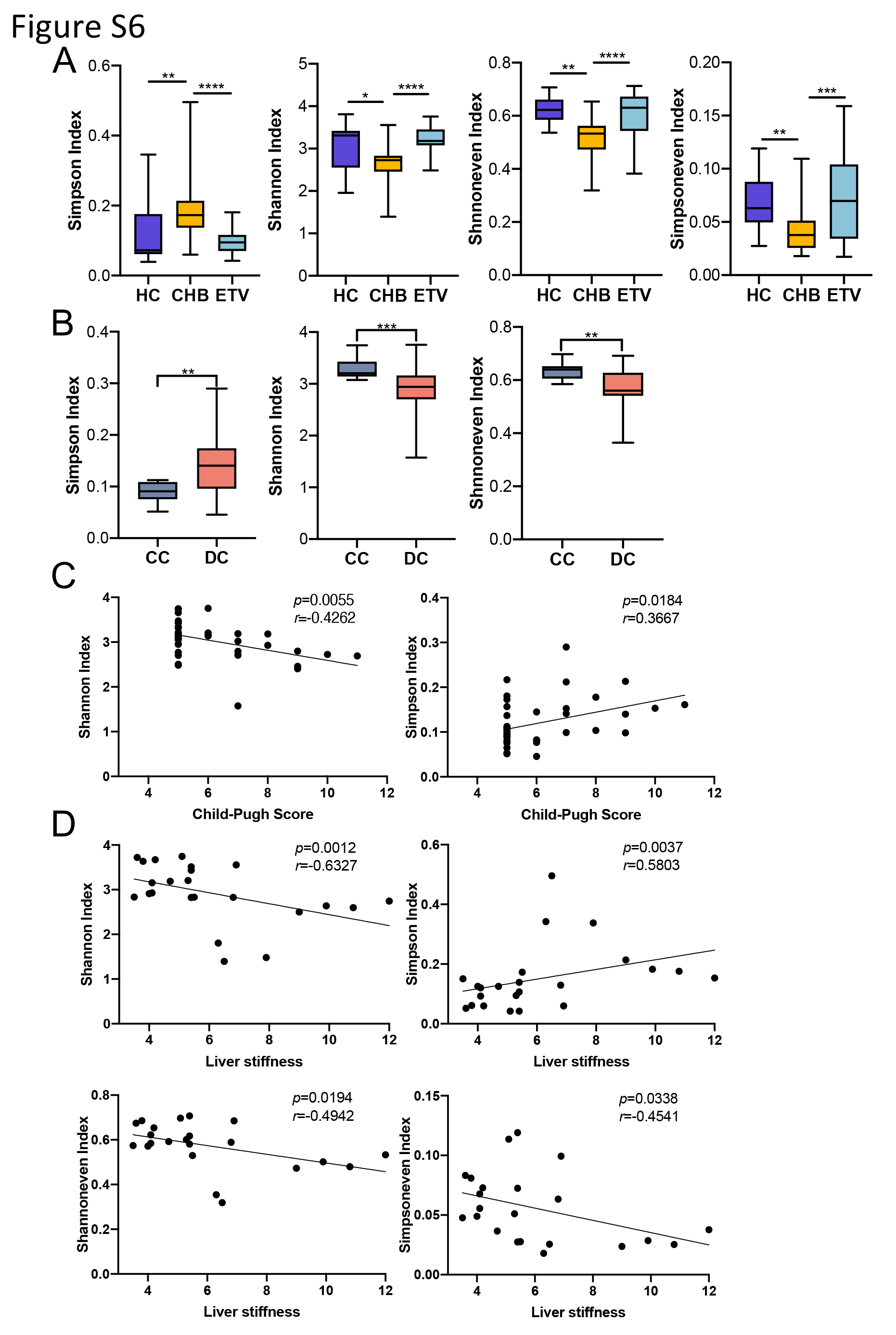

Supplement: Supplemental Material [file KGMI_A_2155018_SM3018.zip › 17 figure S6.png]

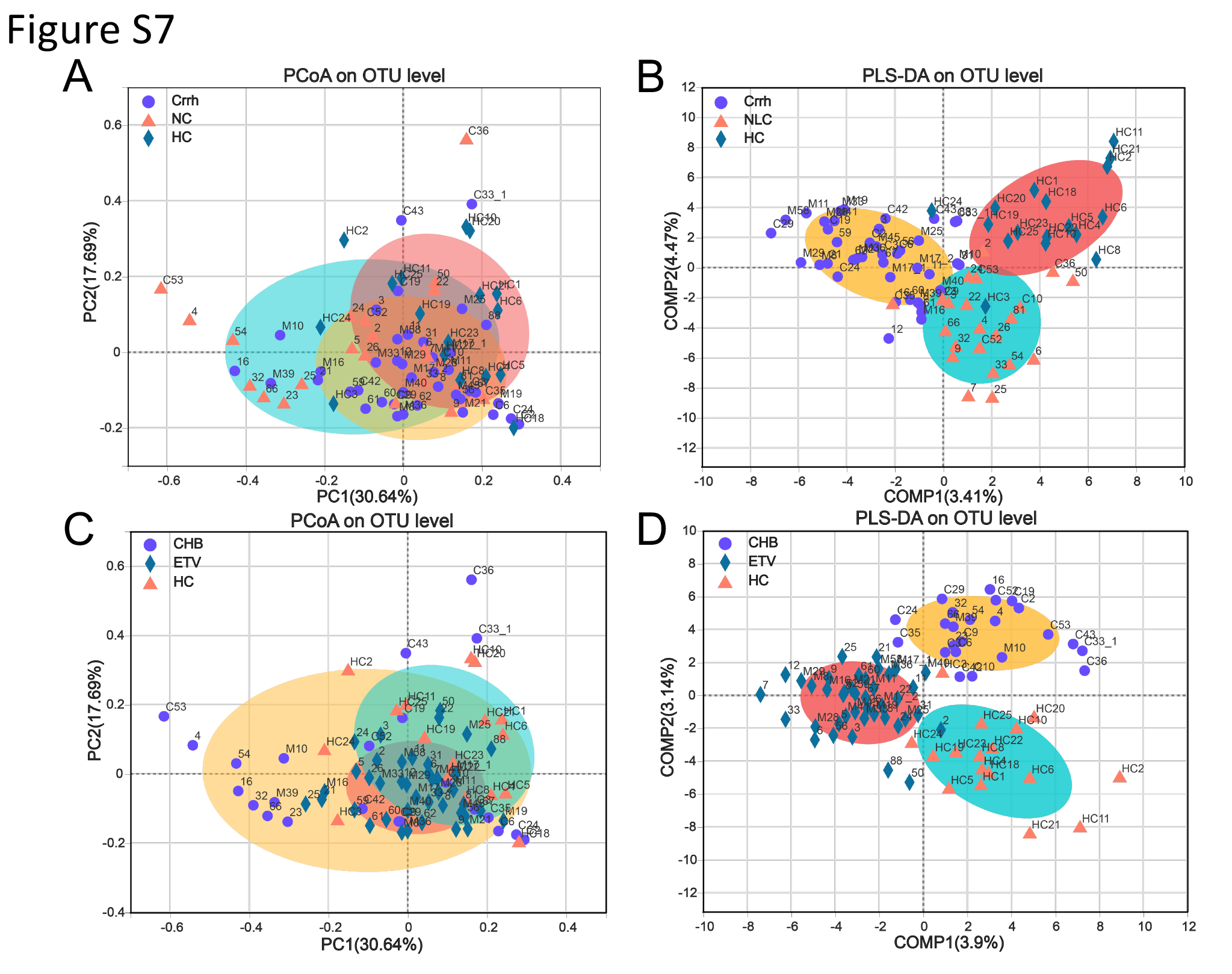

Supplement: Supplemental Material [file KGMI_A_2155018_SM3018.zip › 18 figure S7.png]

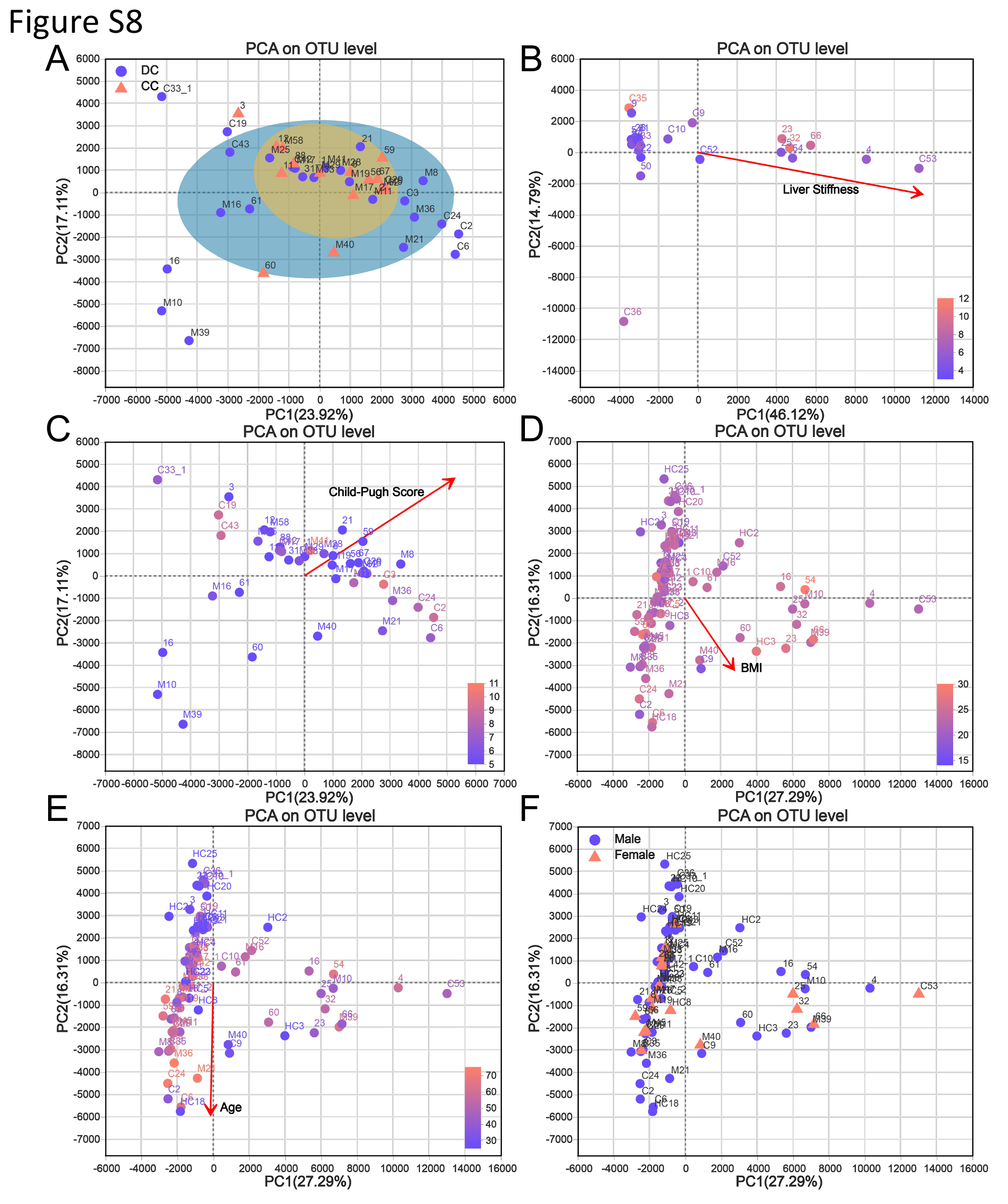

Supplement: Supplemental Material [file KGMI_A_2155018_SM3018.zip › 19 figure S8.png]

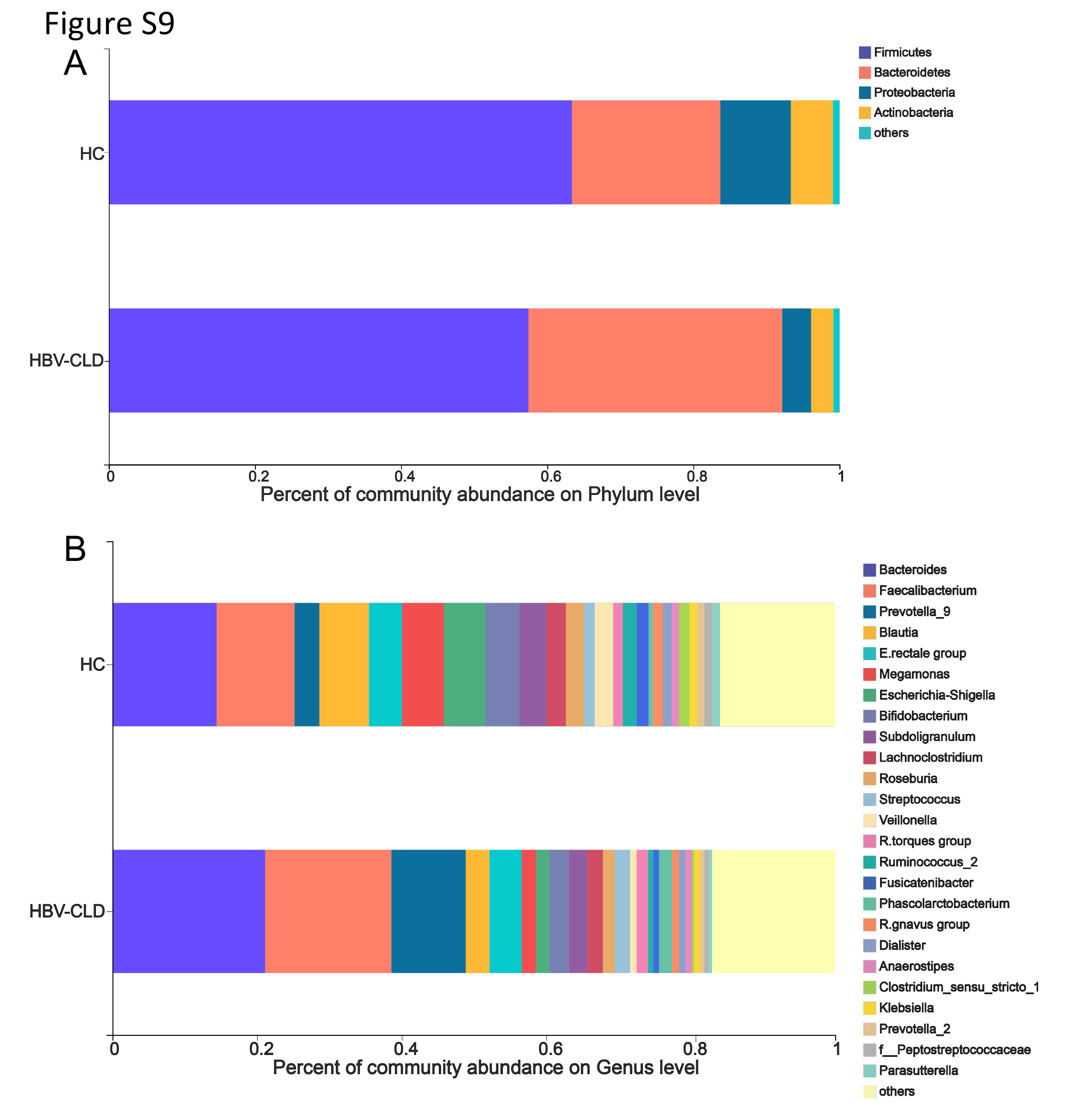

Supplement: Supplemental Material [file KGMI_A_2155018_SM3018.zip › 20 figure S9.png]
